# Nurse Mentors to Advance Quality Improvement in Primary Health Centers: Lessons From a Pilot Program in Northern Karnataka, India

**DOI:** 10.9745/GHSP-D-15-00142

**Published:** 2015-12-15

**Authors:** Elizabeth A Fischer, Krishnamurthy Jayana, Troy Cunningham, Maryann Washington, Prem Mony, Janet Bradley, Stephen Moses

**Affiliations:** ^a^​ IntraHealth International, Chapel Hill, NC, USA; ^b^​ Karnataka Health Promotion Trust, Bangalore, India; ^c^​ University of Manitoba, Department of Community Health Services, Winnipeg, Canada; ^d^​ St. John’s National Academy of Health Sciences, St. John’s Research Institute, Bangalore, India

## Abstract

High-quality care during labor, delivery, and the postpartum period is critically important since maternal and child morbidity and mortality are linked to complications that arise during these stages. A nurse mentoring program was implemented in northern Karnataka, India, to improve quality of services at primary health centers (PHCs), the lowest level in the public health system that offers basic obstetric care. The intervention, conducted between August 2012 and July 2014, employed 53 full-time nurse mentors and was scaled-up in 385 PHCs in 8 poor rural districts. Each mentor was responsible for 6 to 8 PHCs and conducted roughly 6 mentoring visits per PHC in the first year. This paper reports the results of a qualitative inquiry, conducted between September 2012 and April 2014, assessing the program's successes and challenges from the perspective of mentors and PHC teams. Data were gathered through 13 observations, 9 focus group discussions with mentors, and 25 individual and group interviews with PHC nurses, medical officers, and district health officers. Mentors and PHC staff and leaders reported a number of successes, including development of rapport and trust between mentors and PHC staff, introduction of team-based quality improvement processes, correct and consistent use of a new case sheet to ensure adherence to clinical guidelines, and increases in staff nurses’ knowledge and skills. Overall, nurses in many PHCs reported an increased ability to provide care according to guidelines and to handle maternal and newborn complications, along with improvements in equipment and supplies and referral management. Challenges included high service delivery volumes and/or understaffing at some PHCs, unsupportive or absent PHC leadership, and cultural practices that impacted quality. Comprehensive mentoring can build competence and improve performance by combining on-the-job clinical and technical support, applying quality improvement principles, and promoting team-based problem solving.

## Abstract (precis)

Trained nurse mentors catalyzed quality improvements in facility-based maternal and newborn care by: (1) encouraging use of self-assessment checklists and team-based problem solving, (2) introducing case sheets to ensure adherence to clinical guidelines, and (3) strengthening clinical skills through on-site demonstrations and bedside teaching. Inadequate leadership and staffing were challenges in some facilities. Some social norms, such as client resistance to referral and to staying 48 hours after delivery, also impact quality and mandate community mobilization efforts.

## INTRODUCTION

In India, too many women and infants die from causes that are both preventable and easily treatable. The country accounts for 25% of all child deaths and 20% of all maternal deaths globally.^1^^,^^2^ Evidence points to the critical importance of ensuring high-quality care during labor, delivery, and the immediate postpartum period since maternal and child morbidity and mortality are linked to complications that arise during these stages.^3^^,^^4^ While maternal and infant mortality rates vary significantly by state within India, rural women and children often suffer the worst health outcomes.^1^

India launched the National Rural Health Mission (NRHM) in April 2005 to tackle the high burden of maternal, neonatal, and child morbidity and mortality in its rural populations. Now known as the National Health Mission (NHM), the mission has invested in strengthening infrastructure, building capacity of service providers, and ensuring security of essential health supplies.^5^

Within this context, the Sukshema Project, funded by the Bill & Melinda Gates Foundation and implemented by Karnataka Health Promotion Trust (KHPT) and its partners comprising the University of Manitoba, St. John’s National Academy of Health Sciences, IntraHealth International, and Karuna Trust, developed a mentoring intervention to improve the quality of facility-based maternal and newborn care. Mentoring programs have been introduced in other contexts and countries, notably to support HIV/AIDS service provision,^6^^,^^7^ but the mentoring approach has not been widely applied to maternal and newborn health in India. Evidence suggests that performance problems require multifaceted interventions that go beyond one-time training.^8^ Comprehensive mentoring can respond to this need by combining on-the-job clinical and technical support, applying quality improvement principles, and promoting team-based problem solving.

Problems with provider performance often require multifaceted interventions that go beyond one-time training.

**FIGURE 1.**
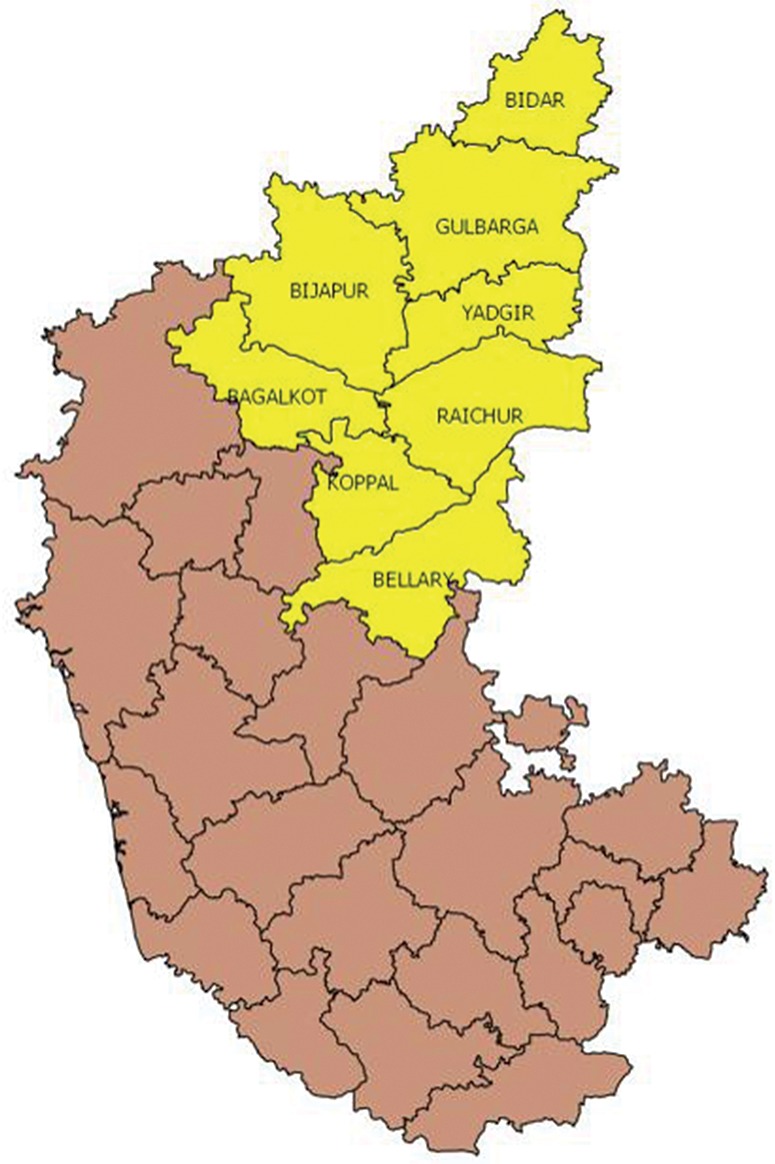
Eight Priority Districts of the Mentoring Intervention, Northern Karnataka, India

The mentoring intervention described in this article focused on the rural poor in 8 priority districts in northern Karnataka that are among the poorest in the state (Figure 1). Although specific district-level data are unavailable, maternal and infant mortality levels in northern Karnataka are known to be higher than state and national averages. As Table 1 indicates, the proportion of institutional deliveries in 2012–2013 was lower in northern Karnataka (80%) compared with the state overall (89%). Nonetheless, the figures represent a marked improvement since 2007, when the government began efforts to increase institutional births. Births taking place in government facilities in Karnataka state rose from 33% to 52% over the 6-year period due to financial incentives provided under the NRHM.^9^^,^^10^

**TABLE 1:** Trends in Institutional Deliveries, Northern Karnataka vs. Karnataka State

	% Institutional Deliveries		% Deliveries in Government Facility	
	2007–08	2012–13	2007–08	2012–13
Northern Karnataka (8 districts)	50.4	79.9	18.2	43.4
Karnataka state	65.1	89.0	33.0	51.8

Source: Regression analysis of data from the District-Level Household and Facility Survey (DLHS)^9^ 2007-08 and 2012-2013 based on methodology by Somayajulu et al.^10^

Given the growing demand for institutional births at government health facilities, the government of Karnataka adopted a strategy to bring maternity services closer to rural populations by upgrading primary health centers (PHCs) and reducing patient volume at higher-level referral facilities. PHCs are now the lowest level of facility expected to provide basic obstetric care in Karnataka.

The success of this close-to-community strategy is predicated on improved availability and quality of PHC services and capacity to manage referrals. In 2011, a situation analysis in the 8 intervention districts identified the need to improve PHC providers’ knowledge and competence in managing maternal and newborn care and to address facility-level factors such as drug stock-outs and lack of infrastructure. Findings revealed that providers did not follow best practices such as active management of the third stage of labor (AMTSL), use of a partograph, or essential newborn care.^11^ Moreover, labor augmentation, although not a recommended practice in facilities without capacity to conduct cesarean deliveries, was found to be very common. Some PHCs also lacked the drugs and equipment needed to provide delivery services and exhibited weak referral and follow-up systems.^11^

This article describes the experience of implementing a quality improvement intervention through mentoring in PHCs. It offers results from a qualitative assessment that identified and described program successes and challenges from the perspective of mentors and PHC teams, including lessons learned and recommendations. The project also conducted a stratified random pre- and post-evaluation of the mentoring intervention that will be described in a future article. It is worth noting that the findings presented in this article are consistent with the findings of the quantitative evaluation.

## THEORY OF CHANGE AND INTERVENTION

Our theory of change of the quality improvement intervention postulated that on-site mentoring would contribute to improved health worker performance and health care quality (including referral management) at PHCs, ultimately leading to better maternal and newborn health outcomes. The mentoring intervention, implemented between August 2012 and July 2014, integrated on-site clinical mentoring with facility-based quality improvement processes to support delivery of critical maternal and newborn care services at PHCs according to government guidelines. The project’s mandate was twofold: (1) to establish evidence of how mentoring affects quality of care, and (2) to demonstrate the process of scaling-up a mentoring program across the region.

The mentoring intervention combined on-site clinical mentoring with facility-based quality improvement processes.

We implemented a peer mentoring model by hiring and training a new cadre of 53 qualified nurses to mentor the PHC staff nurses who provide labor and delivery services. Salaries for the mentors were aligned with government pay scales so the state government could easily absorb them in the future if it chose to sustain the mentoring program.

The nurse mentors were recruited from northern Karnataka through job postings in local papers. Candidates were screened considering criteria such as qualifications, clinical experience, communication skills, abilities and inclination toward teaching and mentoring, and willingness to travel. Most of the candidates applying had 2 years of work experience, and very few had ever undergone training in skilled birth attendance outside their initial training. Almost all the nurse mentors recruited were diploma-level nurses. Most of the mentors had previously worked in small private clinics and hospitals, while 5 had worked as contract nurses in government hospitals prior to joining the project. All the mentors were recruited from within the districts as we wanted to determine if mentoring capacity could be developed locally.

Mentors participated in a 5-week initial training at St. John’s Medical College and Hospital in groups of 15 to 20. Nine trainings were conducted over a 15-month period as districts were phased into the program. The thrust of the training was to equip mentors with the competencies and tools required to support PHC staff in addressing clinical and systems gaps. To this end, the training updated trainees’ clinical skills in labor and delivery as well as in broader quality improvement and mentoring. The trainings were designed to be interactive and competency-based, with opportunities for trainees to learn and practice in the classroom setting using models and skill stations. Trainees also rotated through clinical settings to observe and practice what they learned in the classroom. The training was evaluated using pre- and post-test knowledge and skills tests and objective-structured clinical examinations.

Nurse mentors received a 5-week initial training.

At the conclusion of the training, mentors received backpacks containing reference materials, demonstration models, flip charts, and reporting formats to take on their PHC visits. Quarterly support visits by trainers from partner organizations (both doctors and nurses) were organized to provide further support to nurse mentors in the field. Mentors also participated in clinical postings and semiannual refresher trainings that helped them to gain confidence and competence in their role as mentors.

The mentoring intervention was scaled-up in 385 PHCs in 8 districts. These 385 PHCs provide 30% of total deliveries in northern Karnataka. Two districts served as pilot sites and were also designated for the stratified random pre- and post-evaluation. In these pilot districts, only half of the PHCs received the mentoring intervention for the first year (August 2012–August 2013). (Information from this evaluation is being developed as a separate publication.) During the scale-up phase, we introduced the mentor program in the other 6 districts, 2 districts at a time (October 2012, December 2012, March 2013). By September 2013, soon after the completion of the evaluation, the intervention was also rolled out in the 54 PHCs of the control arm of pilot districts, thus saturating all the 24/7 PHCs in the region.

The mentoring intervention was scaled-up to 385 primary health centers in 8 districts.

The mentors were responsible for mentoring staff in 6 to 8 PHCs within their assigned blocks in the district. Districts comprise an average of 7 blocks, and each PHC is sanctioned to have at least 1 medical officer (MO) and 3 staff nurses as well as other support staff. Typically, mentors lived within their assigned blocks so that their commuting distance was no more than 90 minutes to their facilities each way. Mentors who chose to live in the district headquarters had longer commutes to outlying blocks. In instances where the distance was greater, mentors spent the night at the facility rather than commuting back and forth. Mentors were responsible for organizing their own transport to facilities using common modes of local transportation (e.g., public bus, shared private van, or vehicles known as tempos). Mentors were introduced to the district government officials and supported by project staff in developing visit plans for their allocated facilities. They visited their designated PHCs 6 times in the first year.

The mentors spent 2 to 3 days at the PHC to provide clinical mentoring to staff nurses and also worked with other PHC staff to engage in team building and problem solving for quality improvement using a variety of tools and techniques. After 1 year, mentors adjusted the frequency of their visit schedule based on PHC clinical volume and the level of performance improvement still required. Some high-volume PHCs received monthly visits while PHCs with lower delivery loads received quarterly visits.

**FIGURE 2.**
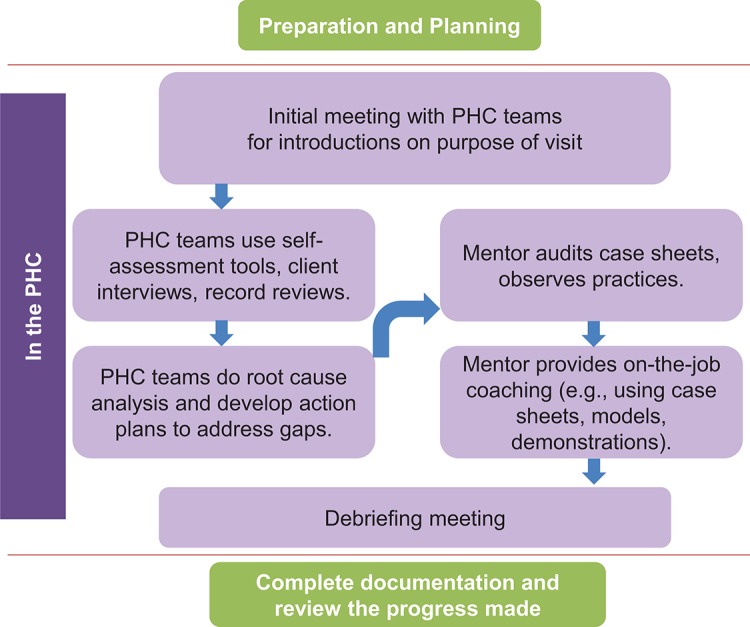
Typical Nurse Mentoring Visit

Figure 2 depicts a typical mentoring visit that begins with mentors explaining the purpose of the visit to PHC staff. Then the mentors work with PHC teams on quality improvement processes and provide clinical support to staff nurses. The mentors conclude the visit with a final debrief meeting with PHC leadership to share progress and action plans. Mentors upgraded PHC provider skills through case reviews, case studies, mini-lectures, demonstrations, modeling of good practice, and bedside case discussions. During deliveries and postnatal care, mentors observed staff practices and provided guidance and assistance as needed. When staff were busy with routine duties, mentors used that time to conduct case sheet audits to identify provider gaps to subsequently address in one-on-one or group coaching. Drawing on quality improvement principles, mentors introduced self-assessment and action planning processes to all PHC staff to promote facility-based quality improvement. In this way, mentors also encouraged a team approach to address specific problem areas (e.g., equipment and supply logistics, infection prevention) and improve referral processes.

After each round of visits, mentors and other project staff met together as a team in each district to share experiences, review monitoring data, submit reports and expenses, and plan for future visits.

## METHODS

The findings in this paper derive from a mix of qualitative methods, including structured observations of 13 mentor visits, 9 focus group discussions (FGDs) with 38 mentors, and 25 interviews (individual and in small groups) with PHC staff and district health officials (Table 2). Mentor interviews, focus groups, and observations were timed to collect information after each round of mentor visits (e.g., first visit, second visit) to fully capture the progression of the intervention over time. Although the study was exploratory in nature, the use of multiple methods allowed for a thorough examination of themes and viewpoints from more than one perspective, providing a rich detailed description and analysis of the mentoring process, generating perceptions about the effectiveness of mentoring on service quality, and yielding suggestions for improving the mentoring program. Certain mentors in the pilot district were also interviewed more than once during the inquiry period to ascertain their perspectives on facility-level changes.

**TABLE 2:** Summary of Qualitative Study Methods

Study Methods	Participants	Number of Sessions/Participants	Purpose
Mentor observations	Trained mentors	• 13 observations • 11 participants (2 observed twice)	Document behaviors of mentors and their interactions with PHC staff
Focus group discussions	Mentors	• 9 focus groups • 56 participants in groups of 6–8 each (including 18 mentors interviewed more than once)	Gather mentor self-assessments and perceptions of the mentoring process and changes in PHCs
Individual and small-group interviews	PHC medical officers, staff nurses, pharmacists, district health officers	• 25 interviews • 25 participants (1–4 per session)	Gather perceptions of the mentoring process and assess mentoring tools and procedures, challenges, successes, and PHC improvements

Abbreviation: PHC, primary health center.

### Data Collection and Analysis

We conducted 5 structured field visits between September 2012 and April 2014 to carry out observations and interviews in 13 PHCs in 6 districts. A senior technical advisor (EAF), not directly involved in the day-to-day operations of the mentoring program, conducted the interviews and observations with support from project staff. Data were collected using project-specific observation checklists and guides for the semi-structured interviews and focus group discussions. Interviews were not taped; however, detailed notes were taken by hand and later electronically transcribed into Microsoft Word. Data were analyzed using grounded theory,^12^ in which themes and subthemes emerge from the coding of text during the process of data reduction and analysis. The analysis captured a wide range of perceptions, represented through both shared and individual viewpoints in the results section below.

## FINDINGS

Findings from the interviews and observations are organized into 3 major themes—program successes, changes in PHC quality, and program challenges—described below in detail and summarized in Table 3.

**TABLE 3:** Key Findings About Nurse Mentoring Program in Northern Karnataka, India, Based on Qualitative Interviews and Observations

Quality of Primary Health Centers: ✓ Areas Improving X Areas Resistant/Slower to Change	Success Factors	Challenges
**Clinical Practices**		
✓ Better understanding and use of AMTSL ✓ Diminished use of labor augmentation ✓ Routine administration of vitamin K ✓ Use of radiant warmers for low birth weight newborns ✓ Better understanding of how to handle complications such as newborn resuscitation and postpartum hemorrhage X Postnatal check-ups at 15-minute intervals difficult to comply with X Objections by MOs who were not updated with latest clinical guidelines	• Rapport and trust between nurse mentors and staff nurses • Strengthening of nurses’ clinical skills through demonstrations, bedside teaching, case sheet reviews, and case studies	• High-volume PHCs require more support than low- or moderate-volume facilities • More difficult for mentors to engage with busy staff nurses • Cultural practices that undermine provision of comprehensive care (e.g., arriving in advanced stages of labor, discharge before 48 hours)
**Availability of Equipment, Drugs, and Supplies**		
✓ Procurement of needed equipment, drugs, and supplies ✓ Creation of MNCH complication kits ✓ Replacement of damaged equipment ✓ Increased availability of drugs and supplies X Deficiencies in basic infrastructure harder to address	• Quality improvement processes and tools, including team-based assessment and action plans • Access to government untied funds to procure needed supplies	• Absent or inadequate PHC (MO) leadership support discouraged teamwork in a small number of facilities
**Referral Processes**		
✓ Improved appropriate identification and pre-referral management ✓ PHCs posted referral directories ✓ Nurses called referral facilities in advance more often ✓ Nurses increasingly tracked referral outcomes X Automatic referral without prior assessment continued in some instances	• Use of case sheets to identify complications requiring referral • Case reviews and mentor reinforcement of referral guidelines	• Understaffed facilities and overworked staff find it hard to perform complete pre-referral management • Inadequate referral facilities • Patient and family member resistance to referral
**Infection Prevention**		
✓ Improved sterilization practices ✓ Greater cleanliness in labor rooms X Overall facility cleanliness still deficient X Deficiencies in basic infrastructure (water, toilets)	• Mentor reinforcement of infection prevention • Demonstration of infection prevention practices with all nurses and cleaners	• Cleaning staff resistant to changing practices • Long time needed to change ingrained attitudes and practices

Abbreviations: AMTSL, active management of the third stage of labor; MNCH, maternal, newborn, and child health; MO, medical officer; PHC, primary health center.

### Program Successes

Nearly all the PHC staff interviewed appreciated the mentoring program and credited it with helping them improve service delivery. One MO stated:

I am very happy with mentoring. We have made a lot of changes since mentors have come, and sisters’ [nurses'] knowledge has increased and they have learned more skills.

Factors contributing to program success included: (1) mentors’ ability to establish trust, (2) promotion of team-based assessment and problem solving, (3) continual strengthening of nurses’ clinical skills, and (4) support for use of case sheets.

#### Building Trusting Relationships

The ability of mentors to build rapport with mentees was crucial to the intervention’s acceptability. Rapport was largely achieved by introducing the mentors first to the district leadership and subsequently to the medical officers of the PHCs at the monthly meeting prior to rollout of mentoring visits. During the monthly meeting, the project’s district program manager described the goals and objectives of the mentoring program and the role of the nurse mentor in improving quality of care within the facilities. In addition, when the visit plans were made, the nurse mentors called the MO to ensure his/her presence at the first visit before actually making the visit. This preparatory work helped build initial rapport, which paved the way for the first visit. Acceptance was enhanced when there was sufficient advance communication with district and PHC leadership before the first mentoring visit about the program and its intent. Without this rapport, it would have been easy for PHC teams to perceive the mentors as outsiders coming in to inspect their facilities and report on them to higher authorities. In certain instances, the introduction at the monthly MO meeting did not take place (for example, when a local strike or other last-minute government priority caused the monthly meetings to be canceled) and only a circular was sent out to the PHCs by the district administration about the mentoring program. In these instances, some of the PHCs were initially reluctant to have mentors visit their facilities.

Mentors reported feeling like they often had to prove themselves in their first visits. In one instance, an MO spent 45 minutes asking a mentor technical questions before giving his PHC staff the green light to learn from her. Nonetheless, in most cases, mentors were able to build trust within the first few visits. As evidence, nurses and other PHC staff praised the mentors’ professionalism and interpersonal skills, describing mentors as “helpful,” “relaxed,” and “cooperative”:

Even if we are rude or stressed because we are busy, [the mentors] don’t react and are always at ease with us, which helps ease the tension.

#### Instilling Quality Improvement Processes

Many facility problems require staff to work together to identify solutions, yet this rarely happened prior to the mentoring intervention. Staff reported previously “working around” problems (such as drug shortages) rather than directly addressing them. Mentors emphasized the importance of service quality and introduced PHC teams to the idea that improvement is everyone’s responsibility.

Mentors reported that, for the most part, PHC staff willingly engaged with mentors in quality improvement meetings, remarking that they had rarely come together as a team before. As one nurse summarized, “The mentoring program has contributed to improved knowledge and better team work.”

To facilitate quality improvement efforts, mentors introduced a set of 8 self-assessment checklists (Box), designed around the principles of patient and provider rights to quality health care, an approach adapted from successful efforts elsewhere.^13^^,^^14^ (See supplementary material for copies of the full self-assessment checklists.) Mentors found that PHC teams were able to use the self-assessment tools to assess their performance, identify problems, and develop solutions. The mentors faced challenges in the beginning when the PHC staff were hesitant to fill out the checklists honestly, fearing the findings would be reported to higher officials; over a 3-month period, however, mentors developed sufficient rapport and trust, and staff saw the payoff from the self-assessment and action planning process as problems were resolved.

Mentors introduced PHC teams to self-assessment checklists to drive quality improvement.

**BOX. Content of Self-Assessment Checklists for Primary Health Centers**

1. Clients’ rights to safe and competent care
2. Providers’ rights to supplies, equipment, and infrastructure
3. Clients’ rights to access services and continuity of care
4. Clients’ rights to infection-free services
5. Providers’ rights to information, training, and development
6. Clients’ rights to privacy, confidentiality, dignity, comfort, and expression of opinion
7. Clients’ rights to information and informed choice
8. Providers’ rights to facilitative supervision and management

See the supplementary material for copies of the full checklists used in the nurse mentoring program in Karnataka, India.

The role of the mentors as facilitators of the quality improvement meetings and self-assessment process was an integral part of the intervention design. It was expected that over time the PHC leaders would begin to drive quality improvement. Participation in the process helped build interest in and appreciation of self-assessment among PHC staff that may contribute to its longer-term sustainability.

In interviews, mentors and PHC staff noted that the process of reviewing and developing action plans had become an entrenched part of the mentoring visits. In several instances, MOs took immediate action to resolve problems identified. In facilities where the leadership position was vacant (unfortunately a common occurrence) or where the MO did not participate fully in the team meetings, problems were less likely to be resolved. In those instances, the lack of leadership also appeared to demotivate staff from making even those improvements that were within their purview to implement.

#### Increase in Knowledge and Skills

Staff nurses credited mentor support with increasing their knowledge and skills in labor, delivery, postpartum care, and newborn care. Nurses pointed out that mentors had helped them become more systematic and thorough in providing care. One nurse stated:

Mentoring has helped in better understanding in a stepwise manner how to conduct deliveries. Having someone explain these steps is very beneficial.

Another nurse commented:

We didn’t know much before, and now the mentor tells us how to do each thing and explains why we do these things. The mentor reminds us about things we forget.

To facilitate clinical capacity building, mentors followed a semi-structured plan during each visit to ensure coverage of critical topics. The mentors used training models (pelvic model and newborn doll) to carry out demonstrations, providing staff nurses with opportunities to practice skills such as newborn resuscitation. Mentors also provided bedside teaching and demonstrations with pregnant and recently delivered PHC clients. This happened more often in high-volume facilities, but, over time, opportunities for bedside mentoring arose in nearly all PHCs. Case sheet reviews and case studies were other primary teaching approaches.

The mentors used training models to carry out demonstrations and provided bedside mentoring.

**Figure:**
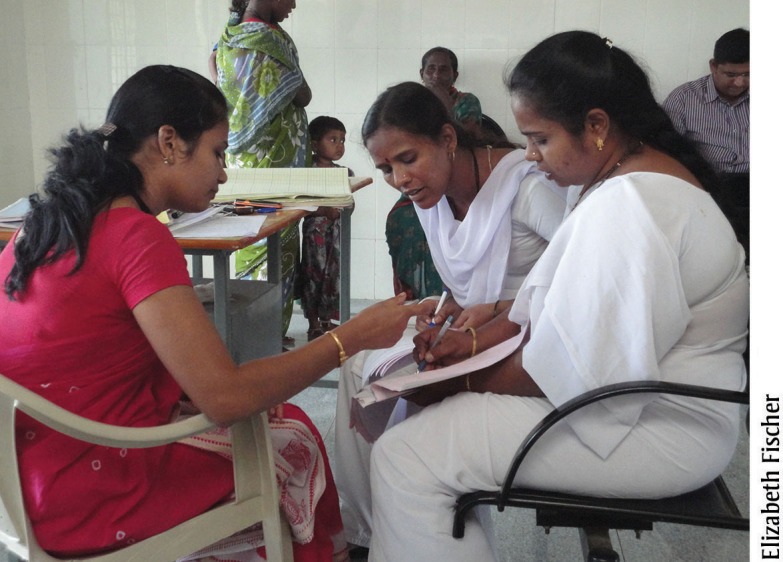
A nurse mentor (left) in Karnataka, India, explains how to complete and use patient case sheets with staff nurses (right).

#### Use of Case Sheets

Given findings of poor provider knowledge of and adherence to government skilled birth attendant (SBA) guidelines, the project introduced a multipurpose case sheet that functioned as a clinical record, job aid, and teaching tool. An existing government case sheet was not well used and lacked explicit guidance on patient care. The mentor-supported case sheet used a checklist concept derived from evidence that checklists can reduce errors and improve compliance with clinical guidelines.^15^ The case sheet included supplemental complication case sheets that guided providers on diagnostic criteria and treatment protocols for complications such as prolonged labor, postpartum hemorrhage, and pre-eclampsia. (See supplementary material for a copy of the case sheet.)

A new case sheet served as a clinical record, job aid, and teaching tool.

As a teaching tool, the case sheets helped mentors focus discussions on compliance with clinical guidelines. The case sheet mechanism also facilitated retrospective case reviews, allowing mentors to refer to cases for teaching purposes despite not being physically present for most PHC deliveries. Finally, the case sheets served as a tool for mentors and PHC staff to monitor changes in quality of care. During each mentoring visit, mentors conducted a case sheet audit and identified and discussed areas for improvement with staff.

Some staff, particularly senior staff nurses, were reluctant to use the case sheets, perceiving them as time-consuming, unnecessary, or a change from long-held practices of not documenting anything. Nurses in busier PHCs also found it challenging to consistently use case sheets as intended in real time and sometimes deferred filling them in until after the delivery. Promoting consistent and correct use of case sheets was, therefore, a major undertaking for the mentors. With continued encouragement, staff became more accustomed to the case sheets, grew to appreciate their value as a job aid, and completed them for most patients.

### Changes in PHC Quality

PHC staff and mentors pointed to noticeable improvements in the PHCs since the mentoring program began. Key areas most often noted included improved clinical practices, improved availability of equipment and supplies, and strengthened referral processes.

**Figure:**
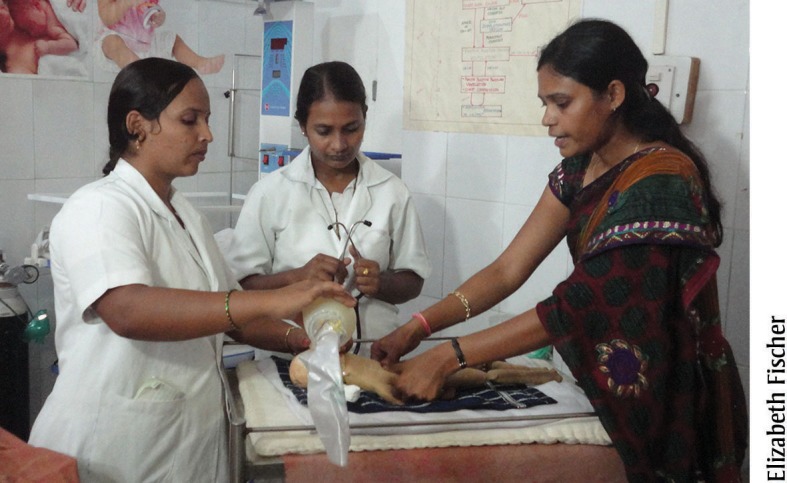
A nurse mentor (right) in Karnataka, India, uses a training model to demonstrate newborn resuscitation to staff nurses.

#### Clinical Improvements

Mentors noted that staff nurses seemed to better understand and practice AMTSL after the first few mentoring visits. Mentors and PHC nurses also reported that the practice of labor augmentation had diminished. Nurses began giving vitamin K more routinely and using radiant warmers for low birth weight newborns. In numerous instances, mentors also were able to intervene and teach nurses how to handle complications. In one case, when a nurse did not know how to do resuscitation with a bag and mask, the mentor stepped in and saved the newborn’s life. In another case, a PHC nurse called a mentor for help with a postpartum hemorrhage situation. Over the phone, the mentor guided her through the steps outlined on the complication case sheet, and the nurse was able to manage and refer the patient. Since then, nurses in that facility have handled postpartum hemorrhage cases on their own.

Active management of the third stage of labor seemed to improve with the mentoring intervention.

Of note, a clinical practice slow to improve was postnatal care. Models of postnatal care provision at the community level in India have demonstrated that simple interventions, such as thermal care and exclusive breastfeeding, can have a significant impact on neonatal survival.^16^ Although these practices are included in the postnatal care guidelines for PHC nurses, mentors noted that postnatal care was often not practiced to standard, and the postnatal care portion of the case sheet was the section most often left incomplete. Both mentors and nurses commented that it was difficult to comply with the guidelines requiring postnatal check-ups at 15-minute intervals, particularly in high-volume facilities with competing demands on nurses’ time. Mentors estimated that only about half of the PHCs communicated postnatal care messages correctly, and they commented that “just reminding staff to give [correct] messages is not enough.” In low-volume PHCs, compliance with postnatal guidelines was easier, as evidenced by one PHC where staff made and posted their own postnatal care charts in the local language.

Despite widespread demonstrated improvements in clinical practice, mentors noted that a small number of PHCs remained “outliers” due to the facilities’ MOs. Mentors described a few MOs who were unwilling or slow to discourage labor augmentation. Similarly, a few MOs objected to the use of vitamin K and would not allow nurses to administer it. Mentors also noted occasional MO concerns about the use of magnesium sulfate. Although nurses provide the vast majority of maternal care at PHCs, they cannot act against MO advice.

Some medical officers were unwilling to change established practices.

#### Improved Availability of Equipment and Supplies

Mentors and PHC staff pointed to improvements in labor rooms and drug supplies as some of the first signs of quality improvement. Many of the changes listed below were instituted by the third or fourth mentoring visit:

- Less congestion and greater cleanliness in delivery rooms
- Replacement of rusty delivery sets
- Replacement or repair of radiant warmers
- Increased availability of oxygen and generators
- Advance preparation of emergency drug kits to ensure availability of drugs and supplies needed for complications

Mentors and PHC staff pointed to improvements in labor rooms and drug supplies as some of the first signs of quality improvement.

As a result of mentoring, most PHCs procured or repaired autoclaves for sterilization. Mentors also trained nurses on practices such as preparing and using chlorine solutions. However, while mentors noted that labor rooms were cleaner and sterilization more common, overall cleanliness in the facilities remained deficient even after multiple visits. Although the mentors involved custodial staff in PHC team meetings and many participated willingly, the substandard levels of hygiene and cleanliness may represent ingrained attitudes and practices that are challenging to change. A combination of organizational and behavioral changes may be required to bring about stronger improvements.^17^

#### Strengthened Referral Processes

Timely access to emergency maternal and newborn services can reduce a significant proportion of maternal and newborn deaths.^17^ Since PHCs have limited ability to provide more than basic obstetric care, strong referral linkages to higher-level first referral units (FRUs) are critical. Fortunately, Karnataka has a relatively well-functioning ambulance service, which makes emergency transportation to referral hospitals less of a barrier than in other parts of India.

Nurse mentors and facility teams promoted interventions to improve referral processes and ensure continuity of care for referred cases, including:

- Using the complication case sheets to identify cases needing referral
- Generating an updated referral directory and referral plan for each facility
- Using referral registers more effectively
- Improving provider communication with referral facilities
- Improving communication with community-based junior health assistants (JHAs) and accredited social health activists (ASHAs) upon discharge to ensure proper follow-up

Mentors and PHC teams reported that referral processes had become more systematic since the start of the mentoring program. According to mentors, most PHCs had posted referral directories. More nurses were calling referral facilities in advance, although some complained that busy district hospitals often left phone calls unanswered. Mentored nurses also increasingly tracked the outcomes of referrals with patients or ASHAs.

Referral processes became more systematic after the mentoring program.

Mentors noted that staff nurses’ ability to identify and handle cases needing referral had improved. One nurse explained, “Earlier we forgot to ask about presenting complaints, but we do so more easily now with the case sheet.” Another nurse reported receiving a call from an obstetrician-gynecologist at a referral hospital praising her for administering magnesium sulfate before referring the patient. Still another nurse described her increased comfort with initial management of complications:

Now we are more confident to manage complications and do referrals. Before, we had knowledge but not confidence.… Now we manage and inform the referral facility and do follow-up.

The general aim of the complication case sheets and the accompanying emphasis on referral management was to decrease automatic referrals of patients without assessment. Improvement in this area remained a work in progress, even after numerous mentor visits. Mentors observed that nurses referred normal deliveries out of PHCs without assessment if patients asked too many questions, if nurses did not want to be disturbed at night, or if patients lacked antenatal care lab work and the nurses did not wish to do the tests. On the other hand, nurses reported that patients often objected to being referred due to perceptions that the referral facility would be less personal, more costly, and more inconvenient. Observations confirmed that providers had to convince families to go to higher-level facilities. Moreover, PHC staff and mentors noted that many FRU-designated hospitals were not able to provide appropriate advanced care because they lacked specialty staff and equipment. As a district health officer conceded:

There will be no use in improving the skills at the PHCs in diagnosing and referring cases if they don’t get good care once referred.

### Program Challenges

The qualitative inquiry identified 4 broad sets of challenges relating to high-volume PHCs, PHC leadership, facility staffing, and cultural practices.

#### Supporting High-Volume PHCs

The project documented wide variation in PHC patient volumes within and across the 8 districts. Just 20 PHCs (5%) accounted for 19% of all monthly deliveries (Table 4). To achieve impact, it was especially important for the project to ensure that those high-volume facilities provided quality services. Yet we learned that busy PHCs require more support than low- or moderate-volume facilities. The mentors noted, for example, that it was more difficult to engage with staff in busy PHCs. Nurses with many patients to treat also were less likely to fill out case sheets or follow protocols. In busy PHCs, mentors had to seize individual teaching opportunities whenever a staff nurse was free, often being disrupted in the process. This meant that the high-volume PHCs needed a more intense focus by the mentors.

Busy facilities required more mentoring support than low- or moderate-volume facilities.

**TABLE 4:** Average Monthly Delivery Volume at Primary Health Centers (PHCs) in Eight Districts in Northern Karnataka, 2013

	Volume Level		
	Low (0–19 deliveries/month)	Moderate (20–39 deliveries/month)	High (≥40 deliveries/month)
No. (%) of PHCs	298 (77%)	67 (17%)	20 (5%)
No. (%) of deliveries	2800 (50%)	1710 (31%)	1060 (19%)

Over time, project staff adjusted the mentoring approach to make the most efficient use of resources and to better target high-volume PHCs. Specifically, the project intensified mentoring support in high-volume PHCs while reducing the frequency (and duration) of mentor visits to quarterly visits rather than bimonthly visits to PHCs that consistently reported low delivery loads. In high-volume PHCs, 2 experienced mentors began to jointly visit the facility for 3 days every month in lieu of every 2 months by a single mentor. The joint visits allowed the 2 mentors to divide up responsibilities and engage with busy PHC staff on the job. Mentors reported observing improvements in staff performance after this revised strategy was implemented. The more frequent visits also helped encourage staff to use the case sheets more often.

Most nurses and MOs in high-volume facilities expressed satisfaction with the more frequent support from 2 mentors. One nurse stated:

Earlier we didn’t give importance to the mentor as we were very busy and it was hard to give attention. Now one mentor can help with outpatient department and labor and the other mentor can teach, so it works much better.

#### Lack of PHC Leadership

Not surprisingly, mentors noted the greatest quality improvement in facilities that had supportive, full-time MOs. Where the MO was indifferent, absent, or available only part-time, the ability of the mentoring program to improve quality of care was limited. In PHCs with inadequate leadership, it proved particularly difficult to encourage a sense of teamwork, as expressed by a nurse in a high-volume PHC who lamented the uncaring attitude of the MO demoralized staff. From this nurse’s perspective, the MO was not interested in improving quality and would not attend group meetings or address identified problems. She noted:

Nobody bothers about us. We ask [the] pharmacist and medical officer for supplies and nothing happens. They don’t agree to sit together to solve problems.

#### Inadequate Facility Staffing

Many PHCs do not operate with the full complement of nurses or other technical staff such as laboratory technicians and pharmacists. PHC staffing guidelines call for 3 nurses to provide 24-hour coverage regardless of patient volume. In the evening and overnight, just 1 staff nurse is on duty. In PHCs that handle high outpatient and delivery volumes, nursing staff are often overstretched and unable to give sufficient time and attention to women in labor or postnatally. Another complicating factor is that it is not uncommon for at least 1 of the 3 nurses to be away for training, marriage leave, maternity leave, or other reasons. One of the PHCs visited was operating with just 1 staff nurse. Understaffed facilities and overworked staff were perhaps most in need of, but least able to, benefit from mentor support.

#### Cultural Practices

Some barriers to improved maternal and newborn outcomes derive from social norms and community practices. Case sheet reviews and interviews indicated that mothers, especially second gravida or more, often come to PHCs only when they are in advanced active labor or fully dilated because they fear automatic referral if they come earlier. However, late arrival limits opportunities to monitor the progress of labor, identify and manage complications, or make timely and needed referrals.

Women delivering in PHCs are expected to remain for 48 hours after delivery as this is the critical window in which maternal and newborn complications most often develop. Interviews suggested that few women remain in the facility that long. The project’s situation assessment identified a number of reasons for shorter stays. First, many facilities lack basic amenities such as functioning toilets, security, or meals. Second, mothers and family members frequently do not understand the rationale for remaining for 48 hours and want to return home to avoid additional transportation costs, to attend to household responsibilities, and to participate in home-based rituals. Even when women remain for the recommended duration, they may not receive postnatal care and support from PHC staff according to guidelines.

Mentors worked with PHC teams to identify and address some of the facility-based barriers discouraging longer stays. Through the team-based quality improvement process, some PHCs arranged for meals and other amenities. However, mentors indicated that these improvements did not appreciably increase the duration of post-delivery stays in any of the PHCs over the course of the mentoring intervention.

## DISCUSSION

The mentoring intervention supported district and facility efforts to improve the quality of care in PHCs to ensure that women coming to this level of facility receive skilled birth attendance and that complications are identified, managed, and referred when needed. The prior situation analysis found many gaps in service quality stemming from providers’ lack of knowledge and skills in conducting normal deliveries or recognizing and managing maternal and newborn complications. Most PHCs also lacked the equipment, supplies, and drugs needed to deliver care according to guidelines. The quality improvement program offered an opportunity to provide tailored support to PHC staff to address these clinical and system-level gaps through on-site mentoring, use of team-based assessment tools and problem solving techniques, and use of case sheets to promote adherence to clinical guidelines.

### Building Capacity of Nurse Mentors

Considerable effort was required to build the mentors’ clinical capacity and expertise, both at the beginning and throughout the project. First, Karnataka had a limited number of senior nurses with the required skills willing to serve as mentors in multiple locations. Many of the nurses who applied and were hired were, therefore, young, relatively inexperienced, and lacked strong clinical backgrounds in maternal and newborn care. Nevertheless, sourcing candidates from the local area was important to demonstrate the scalability and sustainability of a mentoring program. At a professional level, candidates found the new career opportunity of mentor to be interesting and felt they could add value in providing teaching and training—an element of the position that held appeal for many applicants. At the same time, we learned that clinical skills could be taught and that it was critical to recruit outgoing candidates who enjoyed interacting with people; communication skills were harder to instill if not initially present.

Considerable effort was required to build the mentors’ clinical capacity and expertise.

The 5-week mentor training was sufficient to refresh basic knowledge and skills, but mentors needed further on-the-job support and reinforcement through clinical practice and refresher trainings to fully develop their clinical competence and confidence. The project sought to build in a 5-day clinical posting every quarter for all mentors, but finding adequate clinical sites to provide mentors with practical training in labor and delivery posed a significant challenge. This was because hospital staff who did not know the mentors or their level of competence were reluctant to allow trainees to practice their skills. Ultimately, the project developed relationships with a few district and teaching hospitals and rotated mentors through their labor rooms for clinical practice. Each mentor observed 5 deliveries and conducted at least 5 deliveries during the week they were posted, which they recorded in a logbook. High-volume hospitals proved to be a good place to observe complicated obstetric cases, providing mentors with a better understanding of their presentation and management. Unfortunately, these facilities did not always follow correct guidelines and best practices, so mentors were supervised by the technical specialists of the project to help them avoid picking up non–evidence-based practices. The project also supplemented clinical postings with refresher training and on-the-job support from senior clinicians who periodically accompanied mentors on PHC visits to help the mentors grow as effective mentors over time.

### Acceptance of Nurse Mentors by Facility Staff

The mentoring concept is a generally unknown and untested strategy to improve quality of care in India, especially in primary-level facilities. Because of the lack of familiarity with the mentoring concept, we were not sure whether mentors would be accepted by the PHC teams to which they were assigned. The findings from this qualitative assessment furnish positive evidence that the majority of PHC teams interviewed perceived the mentors as helpful. Mentors were able to effectively communicate the purpose of their support in such a way that PHC teams did not feel threatened or blamed. The supportive, collegial tone that mentors brought to their interactions with staff nurses, as well as the respect they showed for MOs, created the basis for forming trusting relationships—an essential ingredient in a successful mentoring program. Because the mentors had no supervisory authority over the PHC teams, nurses were able to respond to them as colleagues rather than authority figures. The perception of mentors as a trusted resource also allowed PHC teams to be open about facility problems.

Nurses and MOs receive preceptor support during the clinical postings that are part of nursing or medical education, but this type of support is not typically available once a health professional is posted to a facility. Moreover, traditional training interventions do not provide follow-up support to ensure transfer of learning once a health worker returns to her workplace. The mentors’ support filled this gap. In fact, many nurses stated that they found mentoring to be better than attending one-time training. The continuity of the mentoring relationship provided the opportunity for ongoing learning and sharing of experiences, and the mentors’ ability to observe and assist nurses over time helped ingrain clinical skills and adherence to guidelines into daily nursing practice. In addition, PHC nurses frequently contacted mentors in between clinic visits to seek advice, confirm diagnoses or treatment decisions, or confer about other issues.

Many nurses thought mentoring was better than one-time training sessions.

### Frequency of Mentor Visits

The frequency of the mentor visits generally seemed adequate and appropriate. The visits were frequent enough to establish relationships and trust, yet spaced apart enough to provide time for staff to practice newly learned skills. The duration of each visit was adjusted over time. Early in the program, it became apparent that a 2-day visit did not allow sufficient time for mentors to cover topics planned for that visit or to interact with all staff given shift schedules and duties. Extending the visits to 3 days afforded mentors extra time to work within the circumstances encountered in each PHC and to reach all staff. We also learned that busy PHCs benefit from more frequent (monthly) visits to instill and reinforce changes in provider practices. Since high-volume PHCs provide a greater contribution to overall deliveries, intensifying support in these facilities can help maximize outcomes. In less busy PHCs, staff had more leisure to absorb and adopt practices into their routines and only required visits every quarter after 1 year of support. To plan a program at this scale, it is important to have guidelines on frequency and duration of visits to manage mentors’ time, but it is also important to allow mentors some autonomy and the ability to adjust visit schedules in accordance with facility circumstances.

### Team-Based Quality Improvement Approach

Although mentors worked most closely with the PHC staff nurses, they were also able to catalyze a team-based approach to improving quality of maternal and newborn care that went beyond what could be accomplished in one-time quality improvement training. The self-assessment guide and action plan tools helped give focus to these team meetings and ensured that the meetings were action-oriented and identified actual problems. It is not clear, however, whether these tools would have been as useful without the facilitation provided by the mentors. On their own, PHC teams tended not to identify deficiencies, but when the mentors probed and asked follow-up questions, team members conceded the need for improvements. Recognizing this tendency for bias, the involvement of a more objective third party such as the mentors appears to contribute to a more effective self-assessment process.^13^

The intervention design required the mentors to prompt PHCs to fill out the self-assessment tools a few times a year. As PHCs made improvements, they identified fewer gaps in subsequent rounds of assessment. Modifying the self-assessment checklists over time might prevent the assessment process from becoming too routine, and additional self-assessment tools could go into more detail on quality standards after basic gaps are addressed. For example, more detailed assessments could address patient-centered care and patient-rights concepts at a deeper level. The fact that PHC teams were comfortable using the self-assessment checklists also suggests that a comparable mechanism could be leveraged to explore issues beyond maternal and newborn care.

### Teaching Models and Tools

Mentors’ ability to use a variety of teaching methodologies helped PHC nurses build their skills and confidence. Nurses appreciated mentors’ effective use of simple models to do demonstrations and observe return demonstrations. Working alongside nurses providing patient care also introduced opportunities for reinforcing guidelines. The mentors proved adept at identifying and taking advantage of teaching opportunities that spontaneously presented themselves but at the same time used the semi-structured mentoring plans to ensure that they covered all critical topics. Additionally, the case sheet proved useful as both a teaching aid and a way for mentors to discuss and review cases. As a job aid, the case sheet provided guidance to nurses for normal and complicated cases. At the same time, introducing a new tool into nurses’ work routines was challenging, and mentors had to continually reinforce the importance and value of the case sheet. The systematic approach to patient assessment and reporting that the case sheets demanded was new to many nurses; some nurses took to it while others were ambivalent and saw it as an added burden. We do not know if case sheet use would continue in the absence of mentors encouraging and monitoring its use. If the case sheet mechanism is adopted by others for use at scale, it will be important to build in adequate training and support structures to help providers use the case sheets as intended. It is not sufficient to simply distribute the case sheets and expect providers to use them.

### Challenges to Improving Quality of Care

Despite a high level of acceptance by most PHCs, mentors’ ability to prompt changes in quality of care was limited by factors beyond their control. For example, mentors’ good intentions could be undermined if the MO was not in favor of the program or the PHC lacked full-time leadership. Instances of MO opposition underscore the need to orient MOs on the latest guidelines and convince them to support adherence to guidelines. Mentors also found it harder to promote changes in PHCs that were understaffed for the volume of patients they handled, leaving staff too busy to engage with the mentors. Mentors cannot fix the underlying problem of staff shortages, a situation that is only likely to improve if the authorities take action to revise their staffing policies and better align number of staff to patient volume. Tools such as the World Health Organization’s Workload Indicators of Staffing Need (WISN) could help identify staffing requirements aligned to workload.^18^^,^^19^

Orientation of medical officers to the latest guidelines could help convince them to support quality improvement practices.

Other factors that stand in the way of improved maternal and newborn health are deeply rooted in cultural and social norms over which mentors and PHC teams have limited influence. Women’s tendency to arrive late in labor, to resist referral if required, and to object to staying 48 hours after delivery are just some examples. There was little evidence, even anecdotally, that improvements in the quality of care provided at PHCs might mitigate these cultural factors. Community-level interventions are needed to raise awareness of the importance of seeking care and recognizing and acting on danger signs. Similarly, the assessment’s findings regarding the complexities of effectively managing and making referrals indicate that both community and health system factors need to be strengthened to fully improve access to quality referral services.

### Scalability and Sustainability of the Nurse Mentoring Approach

Given its dual focus on clinical support and systems strengthening, the nurse mentoring intervention is a comprehensive approach to improving the quality of intrapartum care at the facility level. Mentors work with PHC staff to address the multifaceted processes that must be aligned and integrated within a facility to improve maternal and newborn health outcomes. The nurse mentoring intervention was piloted at sufficient scale and generated evidence of improved quality across the high-priority districts. The scalability of this type of intervention is particularly relevant to Indian and other similar contexts that have shown considerable progress in increasing the percentage of facility-based births. Unless these facilities are able to provide quality care, countries will fall short of achieving improved maternal and newborn health outcomes.^20^^,^^21^ Yet at the same time the public health sector in India faces challenges in filling positions of doctors and specialists, so exploiting the available nursing workforce to improve service quality can be a viable option.

Evidence of the scalability and sustainability of this mentoring approach is promising. In India and globally, more government leaders are becoming champions for quality improvement within government health systems, yet to develop a culture of quality at all levels within government will take time. Public-private partnership models in which governments work with NGOs or professional organizations may be one approach to scaling-up and sustaining a mentoring program. In India, state governments often have the resources through NHM to pursue such models if they choose to do so. For example, the Karnataka experience was adopted in another state of India, Uttar Pradesh, which is implementing the nurse mentoring program in 25 high-priority districts of the state with government funding and donor-provided technical support.

The state of Uttar Pradesh has adopted Karnataka’s mentoring program.

Beyond wholesale adoption of this model, elements of the mentoring program are also incorporated in recent Government of India guidelines that call for the establishment of skill labs and training of nurse mentors to provide on-site mentoring support to trained staff in maternal and newborn care. The most recent national quality assurance guidelines also include aspects of setting-up quality improvement committees within facilities and use of self-assessment and action planning for addressing systems gaps—the same approaches that we found useful in the mentoring intervention. These developments are timely and acknowledge the need for quality improvement processes and on-site support in enhancing provider skills and performance and, ultimately, health outcomes. Growing recognition among India’s health experts on the need for strengthening the capacities of nurses and midwives through ongoing support and mentorship and a focus on quality improvement offers promise for sustaining these interventions within the existing government health system.^22^^-^^25^

## CONCLUSION

Implementation of the mentoring intervention demonstrated the ability of mentors to work with facility-level staff to identify gaps and improve the quality of maternal and newborn services in rural PHCs in northern Karnataka. The mentors used quality improvement processes and tools, such as self-assessment checklists, to encourage PHC teams to drive the improvement process themselves, and they drew on a number of teaching methodologies to strengthen staff nurses’ clinical skills including on-site demonstrations, bedside teaching, and case sheet reviews. The program was implemented at scale in a short period of time, staff were accepting of mentors and the guidance they provided, and mentors demonstrated their ability to increase staff nurses’ capacity and confidence in areas such as active management of the third stage of labor and routine administration of vitamin K. In many PHCs, nurses reported being better able to provide care according to guidelines and to handle maternal and newborn complications. Facilities were also better organized, equipped, and supplied to deliver quality services, and referral procedures improved. Challenges encountered in some facilities included inadequate commitment of senior personnel at the district and PHC levels and inadequate staffing levels. Overall, the evidence gathered holds out the promise that comprehensive mentoring can be an effective intervention for improving the quality of facility-based maternal and newborn care.

## Supplementary Material

Supplementary Material

## Notes

**Peer Reviewed**
