# Supplementary material for: Nurse Mentors to Advance Quality Improvement in Primary Health Centers: Lessons From a Pilot Program in Northern Karnataka, India
Source: Glob Health Sci Pract. 2015 Dec 15;3(4):660–75. doi: 10.9745/GHSP-D-15-00142 (PMC4682589; doi:10.9745/GHSP-D-15-00142)
Supplement: Supplementary Material [file supp_3_4_660__index.html]

Supplementary Material 

# Nurse Mentors to Advance Quality Improvement in Primary Health Centers: Lessons From a Pilot Program in Northern Karnataka, India

## GHSP-D-15-00142 Supplementary Material

Fischer et al. doi: 10.9745/GHSP-D-15-00142

- Supplementary Material - Fischer et al. doi: 10.9745/GHSP-D-15-00142
- Supplementary Material - Fischer et al. doi: 10.9745/GHSP-D-15-00142
