# Supplementary material for: Nurse Mentors to Advance Quality Improvement in Primary Health Centers: Lessons From a Pilot Program in Northern Karnataka, India
Source: Glob Health Sci Pract. 2015 Dec 15;3(4):660–75. doi: 10.9745/GHSP-D-15-00142 (PMC4682589; doi:10.9745/GHSP-D-15-00142)
Supplement: Supplementary Material [file GHSP-D-15-00142_15-00142-Fischer-Supplementary-Material-Case-Sheets.pdf]

Project Sukshema | Department of Health, Government of Karnataka | National Rural Health Mission

MATERNAL AND NEWBORN CASE SHEET FOR USE AT 24x7 PHCs

Project Sukshema supports Karnataka to improve maternal, newborn and child health outcomes in rural populations through the development and adoption of effective operational and health system approaches within the NRHM. The project is implemented in the 8 districts of Northern Karnataka (Bagalkot, Bellary, Bidar, Bijapur, Gulbarga, Koppal, Raichur and Yadgir. The project is currently piloting an intervention in Bellary and Gulbarga districts to improve the quality of delivery and postpartum care at 24x7 PHCs by providing on site mentoring to the staff through a cadre of MNCH Mentors. As a part of the pilot, the project introduces this modified Case Sheet, along with 8 separate Complication Sheets for each of the most commonly diagnosed complications, as a job aid to the staff attending delivery. This job aid aims to help the PHC staff in providing a comprehensive and quality care to the woman during initial assessment, labour, delivery and immediate postpartum period and to the newborn. More specifically, it (1) reminds the sequence of the different steps to be followed at each stage (2) reminds the correct diagnosis of complications (3) reminds the appropriate procedures and drugs for the initial management of complications before referral and (4) facilitates easy and quick documentation for future audits for quality improvement.

General instructions

1. Use ball point (black or blue) for recording on the Case Sheet.
2. Put a √ as appropriate on the boxes provided.
3. Use one case sheet for every woman over 20 weeks gestation visiting the PHC.
4. Read and follow the detailed instructions under each section and sub-section of the Case Sheet.
5. Read and record on all sections of the Case Sheet, either before or during or after an examination or a procedure.
6. Use all related Complication Case sheets if multiple complications are diagnosed.
7. Tear off the pink pages of this case sheet, staple them together with the Complication Sheet used, and send them to the facility where the woman/newborn is referred to.

CASE SHEET FOR PREGNANT WOMEN GREATER THAN 20 WEEKS GESTATION

SECTION 1: INITIAL ASSESSMENT

A.FACILITY IDENTIFICATION

District ______________________ Taluka ______________________ PHC Location ______________________

B.BACKGROUND INFORMATION

Name______________________________

Husband's name ______________________

Age ☐☐ yrs

BPL Y ☐ N ☐ Thayi card available Y ☐ N ☐

Thayi card number ☐☐☐☐☐☐☐☐

Address ______________________

Date of arrival (day month year) ☐☐☐☐☐☐☐☐

Time of arrival ☐☐ hrs ☐☐ mins

Date of initial assessment (day month year) ☐☐☐☐☐☐☐☐

Time of initial assessment ☐☐ hrs ☐☐ mins

Contact number ☐☐☐☐☐☐☐☐☐☐

C. PRESENTING COMPLAINTS (Put a √ on Y box if present and a √ on N box if not present, for each complaint)

Fever Y ☐ N ☐

Swelling of face Y ☐ N ☐

Headache Y ☐ N ☐

Blurred vision Y ☐ N ☐

Vomiting Y ☐ N ☐

Fits/seizures Y ☐ N ☐

Difficulty in passing urine/ less urine Y ☐ N ☐

Palpitation Y ☐ N ☐

Severe weakness / tiredness Y ☐ N ☐

Breathlessness at rest or on mild exertion Y ☐ N ☐

Pain in abdomen Y ☐ N ☐

Contractions Y ☐ N ☐

Watery discharge per vagina/rupture of membranes Y ☐ N ☐

Bleeding per vagina Y ☐ N ☐

Foul discharge per vagina Y ☐ N ☐

Decreased / No foetal movement Y ☐ N ☐

Any other (specify) ______________________

D. MENSTRUAL AND OBSTETRIC HISTORY (Ask the woman and/or refer to Thayi Card)

G ☐ P ☐ A ☐ L ☐ Cycles Regular ☐ Irregular ☐ Length of the cycle ☐☐ days

LMP day ☐☐ month ☐☐ year ☐☐☐☐ EDD day ☐☐ month ☐☐ year ☐☐☐☐

Gestational age ☐☐ wks ☐☐ days Gestational type Single pregnancy ☐ Multiples ☐

# of ANC checkups (Record '00' if none) ☐☐

E.PREVIOUS OBSTETRIC HISTORY (Record particulars for each pregnancy outcome in separate lines. If primigravida, skip to section F)

Year of delivery/ abortion	Mode of delivery (Record NORMAL, INSTRUMENTAL, CAESAREAN, SPONTANEOUS ABORTION, or MEDICAL TERMINATION OF PREGNANCY)	Place of delivery/MTP (Record SC, PHC, CHC, TH, DH, PVT, HOME, or OTHER)	Complications during pregnancy/ delivery/postpartum (Record NONE, APH, PIH, PRE-ECLAMPSIA, ECLAMPSIA, PRE-TERM LABOUR, PRE-TERM RUPTURE OF MEMBRANES, FOETAL DISTRESS, ABNORMAL PRESENTATION, FEVER, PPH, and OTHER (specify)	Outcome (Record ABORTION, STILL BIRTH, LIVE BIRTH, NEONATAL DEATH, INFANT DEATH)

F. OTHER HISTORY

Medical history Diabetes Y ☐ N ☐ Anaemia Y ☐ N ☐

Hypertension Y ☐ N ☐ Heart disease Y ☐ N ☐ Other Y ☐ N☐

(specify) ______________________

Surgical history ______________________

Any allergies Y ☐ N ☐ If yes, specify______________

Medications during this pregnancy

Injection TT given 1 ☐ 2 ☐ Booster ☐ None ☐

Taking any medications Yes ☐ No ☐ If yes, IFA ☐

Other ☐ (specify) ______________________

G. INVESTIGATIONS DONE DURING THE CURRENT PREGNANCY (Record Y or N if the test is already done, based on the Thayi Card/ self report. For each test, record the results and date in respective columns. Determine if tests need to be done/ repeated and record the test results)					
Test	Already done	Date	Result	Done today	Result
Haemoglobin	Y ☐ N ☐			Y ☐ N ☐	
Blood group/Rh	Y ☐ N ☐			Y ☐ N ☐	
RPR/VDRL	Y ☐ N ☐			Y ☐ N ☐	
HIV	Y ☐ N ☐			Y ☐ N ☐	
HbsAg	Y ☐ N ☐			Y ☐ N ☐	
Urine for protein	Y ☐ N ☐			Y ☐ N ☐	
Urine for detection of infection	Y ☐ N ☐			Y ☐ N ☐	
Oral glucose tolerance test	Y ☐ N ☐			Y ☐ N ☐	
Ultrasound	Y ☐ N ☐			Y ☐ N ☐	
Other_______________	Y ☐ N ☐			Y ☐ N ☐	

H.EXAMINATION

General Examination

Weight [|] kgs Height [| |] cms Pulse [| |] beats/min

Blood pressure [| |] / [| |] mmHg Temperature [|] ^{0}C Pallor Y ☐ N ☐ Oedema Y ☐ N ☐

Jaundice Y ☐ N ☐ Lungs clear Y ☐ N ☐ Heart sounds normal Y ☐ N ☐ Any murmurs/additional sounds Y ☐ N ☐

Abdominal examination

Fundal height [|] wks Presentation Cephalic ☐ Transverse ☐ Breech ☐ Other ☐

Contractions present Y ☐ N ☐ If yes, record date and time of start ______________________________ AND

frequency [ ] Number per 10 mins AND Intensity Mild (<20 secs) ☐ Moderate (20-40 secs) ☐ Strong (>40 secs) ☐

Is uterus tender? Y ☐ N ☐ Previous caesarean scar Y ☐ N ☐ If yes, type Vertical ☐ Transverse ☐ Unsure ☐

FHR [| |] beats/min Other findings____________________________________

Vaginal examination (DO NOT perform PV examination (a) if membranes are ruptured and the woman is not in labour or (b) if she has antepartum bleeding and the placental location is not known or if placenta is known to be praevia or low-lying)

Cervical dilatation [|] cms Cervical effacement [| |] % Status of membranes Intact ☐ Ruptured ☐

If ruptured, record date and time of rupture ____

Amniotic fluid visible Y ☐ N ☐ If yes, colour of amniotic fluid Clear ☐ Bloody ☐ Meconium stained ☐

Is there a purulent vaginal discharge? Y ☐ N ☐

Presenting part Vertex ☐ Breech ☐ Shoulder ☐ Cord ☐ Other ☐ _________________ Station of presenting part [|]

Time when examinations were complete [|] hrs [|] mins Pelvic adequate Y ☐ N ☐

I.OVERALL INITIAL ASSESSMENT (Tick a, b, c, d, e or f. Tick all complications observed)

a. Woman not in labour, died ☐ } → Complete Outcomes Sheet and other documentation
b. Woman in labour, died ☐ }
c. Woman not in labour, no complications ☐ → Send home
d. Woman in labour, no complications ☐ → Admit and go to the next section of Case Sheet
e. Woman not in labour, with complications ☐
- Rupture of membranes > 12 hrs ☐ → Manage and refer using Complication Sheet A
- Pregnancy induced hypertension ☐ Pre-eclampsia ☐ Eclampsia ☐ → Manage and refer using Complication Sheet B
- Antepartum haemorrhage with or without shock ☐ → Manage and refer using Complication Sheet C
- Infection with or without shock ☐ → Manage and refer using Complication Sheet D
- Preterm rupture of membranes without preterm labour ☐ → Manage and refer using Complication Sheet E
- Severe Anaemia ☐ Foetal distress ☐ No foetal movement ☐ / Other ☐ (specify) _________________ } → Refer using Complication Sheet H

f. Woman in labour, with complications ☐
- Rupture of membranes > 12 hrs ☐ → Manage and refer using Complication Sheet A
- Pregnancy induced hypertension ☐ Pre-eclampsia ☐ Eclampsia ☐ → Manage and refer using Complication Sheet B
- Antepartum haemorrhage with or without shock ☐ → Manage and refer using Complication Sheet C
- Infection with or without shock ☐ → Manage and refer using Complication Sheet D
- Preterm labour with or without premature rupture of membranes ☐ → Manage and refer using Complication Sheet E
- Severe Anaemia ☐ Foetal distress ☐ No foetal movement ☐ / Mal-presentation ☐ Other ☐ (specify) _________ } → Refer using Complication Sheet H

Date diagnosis made [| | | | |] Time diagnosis made [|] hrs [|] mins

Diagnosed by SN ☐ MO ☐ Other ☐ Was woman admitted? Y☐ N ☐ Time of admission [|] hrs [|] mins

Registration # [| | | |] Name of the staff ______________________ Signature _____________________

SECTION 2: LABOUR MONITORING

A. LATENT PHASE (Start recording as soon as the woman is in labour and dilatation is < 4 cms. Each box corresponds to 60 mins. Record time in hours and minutes corresponding to observation)												
Measure and record foetal heart rate every hour and record the time and the readings below												
Time (hrs and mins)												
FHR (beats per min)												
Measure the following once every 4 hours and record the time and readings.												
Cervical dilatation (cms)												
Maternal pulse (beats per min)												
Blood pressure (mmHg)												
Temperature (Celsius)												

B. ACTIVE PHASE (Start at 4 cms or more dilatation and record all values. Each box corresponds to 30 mins. Record time in hours and minutes corresponding to observation. Plot all these values on the partograph)																				
Measure and record maternal BP, temperature and dilatation every 4 hours or more if necessary. Record everything else half hourly																				
Time (hrs and mins)																				
Cervical dilatation (cms)																				
Contractions per 10 mins																				
Contractions duration (secs)																				
Maternal pulse (beats per min)																				
Blood pressure (mmHg)																				
Temperature (Celsius)																				
FHR (beats per min)																				
Status of amniotic fluid (I for Intact membranes, C for Clear, B for Bloody and M for Meconium)																				
Remarks																				

REVIEW ALL DATA ABOVE ALONG WITH THE PARTOGRAPH TO DETERMINE IF THERE ARE ANY COMPLICATIONS

If there is prolonged rupture of membranes (more than 12 hrs), there is **possibility of infection.**
Graph A: If the plotted cervical dilatation line is to the right of Line A, **labour is prolonged** or **obstructed.**
Graph B: If the contractions do not increase in frequency and duration, **labour is prolonged** or **obstructed.**
Graph C:

- If the maternal pulse is ≥100/min, there is a **possibility of infection or haemorrhage**
- If the maternal BP is ≥140/90 mmHg, there is a **pregnancy-induced hypertension** or possibility of **pre-eclampsia** or **eclampsia**
- If the maternal BP systolic is <90 mmHg, the **woman could be in shock**
- If the maternal temperature is ≥38º C, there is **possibility of infection.**

Graph D:

- If the FHR is <120 beats/min or >160 beats/min, **foetus is in distress.**
- If there is meconium and/or blood-stained amniotic fluid, **foetus is in distress** and there is a **possibility of infection or haemorrhage**

C. OVERALL LABOUR ASSESSMENT (Tick either a, b or c. Tick all complications observed)

a. Woman in labour, died ☐ → Complete Outcomes Sheet and other documentation
b. Woman in labour, no complications ☐ → Go the next Section of this Case Sheet
c. Woman in labour, with complications ☐
- Rupture of membranes > 12 hrs / prolonged / obstructed labour ☐ → Manage and refer using Complication Sheet A
- Pregnancy induced hypertension ☐ Pre-eclampsia ☐ Eclampsia ☐ → Manage and refer using Complication Sheet B
- Antepartum haemorrhage with or without shock ☐ → Manage and refer using Complication Sheet C
- Infection with or without shock ☐ → Manage and refer using Complication Sheet D
- Foetal distress ☐
- No foetal movement ☐ Other ☐ (specify) ____________________ } → Refer using Complication Sheet H

Date diagnosis made | | | | | | | Time diagnosis made | | | hrs | | | mins

Diagnosed by SN ☐ MO ☐ Other ☐ Name of the staff ____________________ Signature ____________________

PARTOGRAPH FOR USE AT PHC (Start at 4 cms dilatation or more; plot always on vertical lines)

Name ______________________________________ PHC registration #___________________________ Parity________________
Date and time of rupture of membranes____

Graph A: Labour (Start plotting dilatation at 4 cms or more with an X on Line A, corresponding to the cervical dilatation at first evaluation. Note the time at the top, corresponding to the first plot. Measure and plot with an X every four hours. If the plotted line is to the right of Line A or B, take action at PHC (manage at PHC and refer) or FRU respectively)

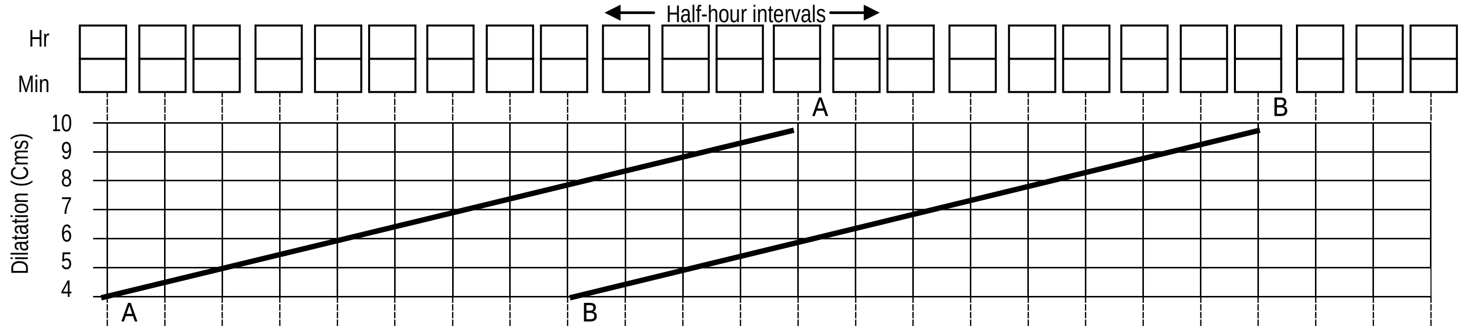

Graph B: Labour (Count contractions for 10 mins every half hour and record with an X; record duration in seconds)

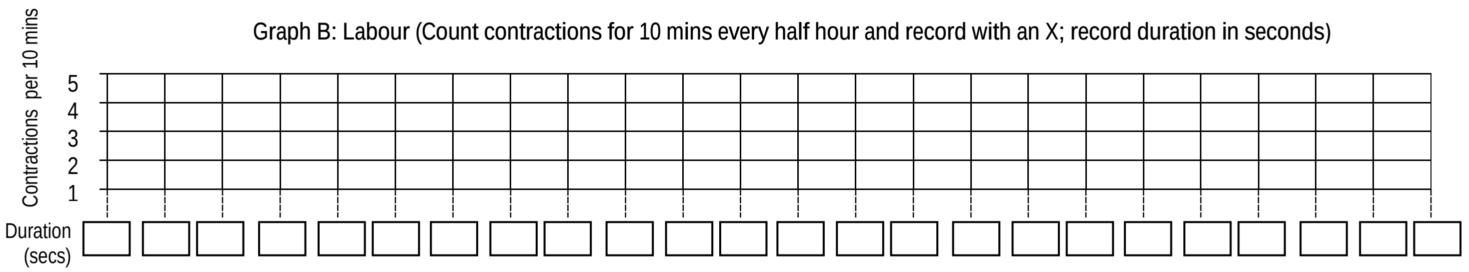

Graph C: Maternal condition (Record pulse with an X every half hour; record BP every four hours using ↕ symbol, the top arrow denoting systolic BP and the lower arrow denoting diastolic BP; record temperature every four hours)

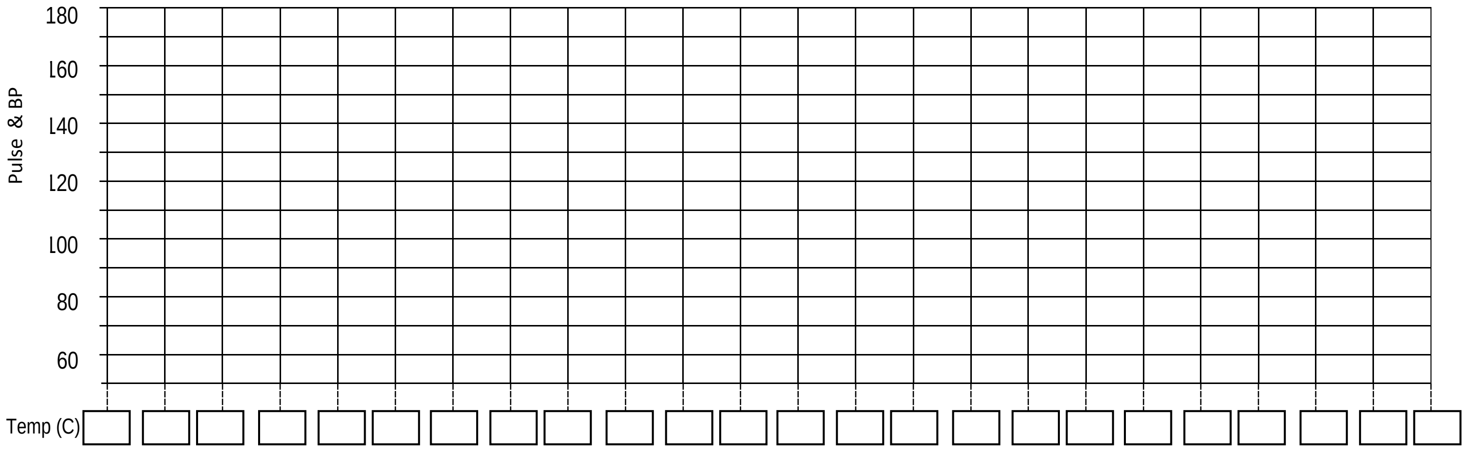

Graph D: Foetal condition (Record FHR with an X every half hour; record amniotic fluid (I, C, B, M) every half hour)

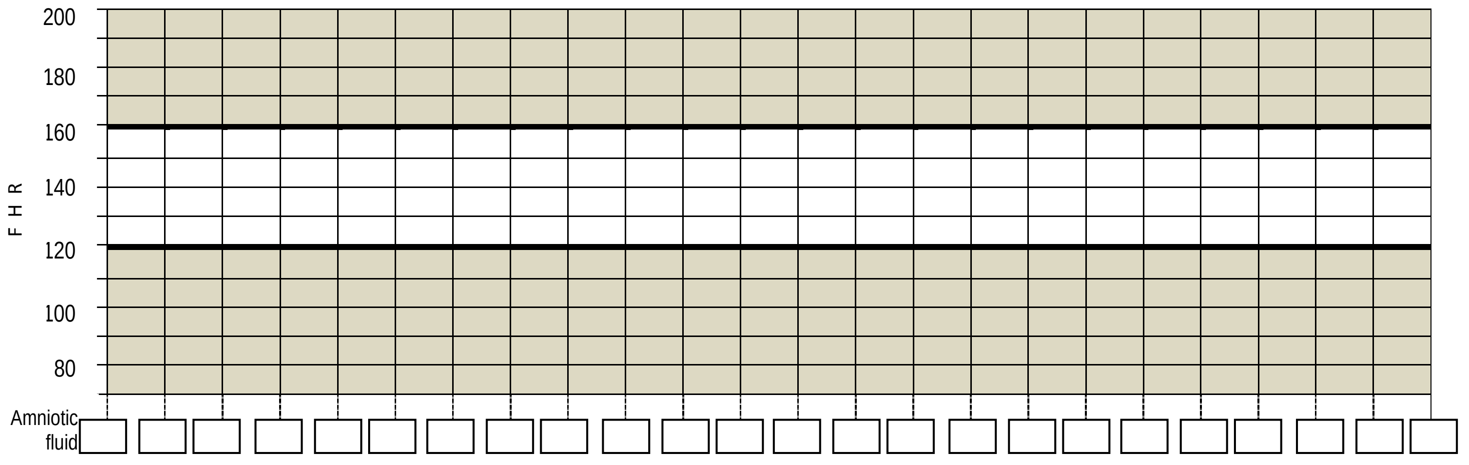

SECTION 3: DELIVERY NOTES

DELIVERY NOTES (Note the particulars for women delivering at the facility. Fill separate forms for twin deliveries)

A. PARTICULARS OF DELIVERY

Date of delivery day [ ][ ] month [ ][ ] year [ ][ ][ ][ ] Time [ ][ ] hrs [ ][ ] mins

Mode of delivery Spontaneous cephalic □ Instrumental delivery □ Breech □ Amniotic fluid Clear □ Meconium stained □ Bloody □

Duration of rupture of membranes [ ][ ] hrs [ ][ ] mins

THIRD STAGE OF LABOUR

Active Management Uterotonic administered Y □ N □ Controlled cord traction Y □ N □ Uterine massage Y □ N □

Uterotonic given either Oxytocin 10 IU IM (preferred) □ or IV □ **or** Misoprostol 600mcg Rectally □ or Orally □ **or**

Methergine 0.2 mg IM □ or IV □ Other ______________ □ If yes, specify Dose_________ Route___________

Placenta Complete □ Incomplete □ Expulsion of placenta Spontaneous □ Curettage □ Manual removal □

Perineum Episiotomy Y □ N □ Perineum tear Y □ N □ If yes, 1st degree □ 2nd degree □ 3rd degree □ 4th degree □

Repair done Y □ N □

Estimated blood loss Average (less than 1 pad in 5 mins) □

Increased (1 pad in 5 mins or continuous bleeding for 10 mins) □ → Manage and refer using Complication Sheet F

Delivery conducted by Name ____________________________________ Signature__________________________________

Designation SN □ MO □

B. PARTICULARS OF THE BABY

General

Sex Male □ Female □ Weight [ ][ ][ ][ ] gms LBW (<2500 gms) Y □ N □

Outcome Live birth □ Still birth □ If still birth, Fresh □ Macerated □ Maturity Preterm(<37 wks) □ Term (37 to 42 wks) □

Post term (>42 wks) □ Apgar score [ ][ ] at 1 min [ ][ ] at 5 mins

Any anomalies Y □ N □ If yes, list __________________________

Resuscitation

Suction done Y □ N □ Oxygen given Y □ N □ Baby breathing after 30 secs Y □ N □

If no, use bag and mask for 30 secs Y □ N □ Baby breathing after another 30 secs Y □ N □

If still not breathing (Asphyxia), continue bag and mask ventilation Y □ N □ Oxygen given Y □ N □

Chest compressions done (if trained) Y □ N □ and Manage and refer using Complication Sheet G

Medication given

Injection Vitamin K Y □ N □ Dose 1 mg for > 1500 gms □ or 0.5 mg ≤ 1500 gms □ Others □ (specify)__________________

Breastfeeding initiation

Time of initiating breastfeeding [ ][ ] hrs [ ][ ] mins

Initiation of immediate skin to skin contact Y □ N □ Chlorhexidine applied on cord Y □ N□

Baby attended by Name ____________________________________ Signature__________________________________

Designation SN □ MO □ Other □

COMPLETE THE CHILD SECTION OF THAYI CARD

C. FOURTH STAGE OF LABOUR

(After delivery, measure and record every 15 minutes for 2 hours for the mother except temperature that should be recorded twice. Measure and record every 15 minutes for one hour and then at two hours for the newborn weighing between 1800 and 2500 gms or that needed resuscitation. For other newborns measure once at the end of each hour. Each column represents 15 mins)

Time (hrs and mins)								
Maternal temperature (in Celsius)								
Maternal pulse (beats per min)								
Maternal blood pressure (mmHg)								
Uterus contracted? (Y or N)								
Bleeding PV (AVERAGE, HEAVY or VERY HEAVY)								
Newborn feeding well (Y or N)								
Newborn colour (PINK, BLUE or PALE)								
Newborn nasal flaring (Y or N)								
Newborn grunting (Y or N)								
Newborn chest retractions (Y or N)								
Newborn respiratory rate (breaths per min)								
Newborn heart rate (Record beats per min)								
Cord bleeding (Y or N)								
Remarks								
Action taken								

D. POST-DELIVERY ASSESSMENT OF MOTHER (Tick a, b or c. Tick all complications observed)	
a. Woman died during delivery ☐	→ Complete Outcomes Sheet and other documentation
b. Woman healthy ☐	→ Go to the next Section of this Case Sheet
c. Woman with complications ☐	
Pregnancy induced hypertension ☐ Pre-eclampsia ☐ Eclampsia ☐	→ Manage and refer using Complication Sheet B
Infection with or without shock ☐	→ Manage and refer using Complication Sheet D
Postpartum haemorrhage ☐	→ Manage and refer using Complication Sheet F
Other ☐ (specify) ________________	→ Refer using Complication Sheet H

E. POST-DELIVERY ASSESSMENT OF NEWBORN (Tick a, b, c or d. Tick all complications observed)	
a. Still birth ☐	→ Complete Outcomes Sheet and other documentation
b. Newborn died ☐	→ Complete Outcomes Sheet and other documentation
c. Newborn healthy ☐	→ Go to the next Section of this Case Sheet
d. Newborn with complications ☐	
Asphyxia ☐ LBW requiring referral (<1800 gms) ☐ Preterm ☐<br>Birth anomalies ☐ Infection / Sepsis ☐ Other ☐ (specify) ________	→ Manage and refer using Complication Sheet G

Date diagnosis made ☐☐☐☐☐☐ Time diagnosis made ☐☐ hrs ☐☐ mins

Diagnosed by SN ☐ MO ☐ Other ☐ Name of the staff ________________ Signature ________________

SECTION 4: POSTPARTUM PERIOD

A.MATERNAL ASSESSMENT (Do the following assessments every 6 hours or more frequently if necessary. Each column represents 6 hours. For some items, ask the woman and for some observe, examine or measure)								
Postpartum day	Day 1				Day 2			
Time (hrs and mins)								
Fever (Y or N)								
Bleeding PV (AVERAGE, HEAVY or VERY HEAVY)								
Headache/ blurred vision (Y or N)								
Breast pain (Y or N)								
Abdominal/ perineal pain (Y or N)								
Vomiting (Y or N)								
Other complaints (specify)								
Temperature (Celsius)								
Pulse (beats per min)								
Blood pressure (mmHg)								
Respiratory rate (breaths per min)								
Uterine tone (HARD or SOFT)								
Uterine height (ABOVE or BELOW UMBILICUS)								
Uterine tenderness (TENDER or NOT TENDER)								
Episiotomy/ tear (PRESENT & HEALTHY or PRESENT & UNHEALTHY)								
Lochia colour (RED, YELLOW, or WHITE)								
Lochia odour (NORMAL or FOUL SMELLING)								

B.NEWBORN ASSESSMENT (Do the following assessments every 6 hours or more frequently if necessary. Each column represents 6 hours. For some items, ask the woman and for some observe, examine or measure)								
Postpartum day	Day 1				Day 2			
Time (hrs and mins)								
Meconium passed in 24 hours (Y or N)								
Urine passed (Y or N) (should be in 48 hours)								
Cry (NORMAL, WEAK, or EXCESSIVE/ IRRITABLE)								
Feeding (WELL or NOT WELL)								
Colour (PINK, BLUE or PALE)								
Nasal flaring (Y or N)								
Grunting (Y or N)								
Chest retractions (Y or N)								
Respiratory rate (breaths per min)								
Heart rate (beats per min)								
Eyes (NORMAL or ANY DISCHARGE)								
Skin (NORMAL, YELLOW or PUSTULES)								
Cord (HEALTHY or UNHEALTHY)								
Activity (ACTIVE/ ALERT, LETHARGIC or UNRESPONSIVE)								
Touch temperature (TOO WARM or WARM or COLD PERIPHERIES / HYPOTHERMIC								
Temperature (Celsius)								
Other concerns (specify)								
Remarks								
Action taken								

C.COUNSELING (Check if provided counselling on the following topics at some points during the PHC stay, not necessarily at one time)			
Postpartum day		Day 1	Day 2
Feeding the newborn			
Warmth for the newborn			
Bathing of the newborn			
Cord care: Nothing on cord			
Immunization for the newborn			
Care of skin/ eyes for the newborn			
Maternal nutrition			
Family planning			
Maternal danger signs	Fever		
	Convulsions		
	Blurred vision/severe headache		
	Increased PV bleeding		
	Foul PV discharge or odour		
	Breathing difficulty		
	Swollen/ red /tender breasts		
	Pain/ difficulty in passing urine		
	Worsening abdominal pain		
	Worsening perineal pain		
Newborn danger signs	Breathing difficulty		
	Feeding problems		
	Convulsions		
	Diarrhoea/vomiting		
	Hypo/hyperthermia		
	Icterus/ yellow skin		
	Stiff (body arched) or floppy		
	Irritability/ lethargy		
	Pustules on skin or boil		
	Not passing urine at least 6 times a day		
	Pus/ inflamed red umbilicus		
	Blood in stool		
Remarks			
Action taken			

D. MEDICATIONS AND IMMUNIZATIONS GIVEN FOR THE NEWBORN (Record if provided during the PHC stay)

BCG ☐ OPV ☐ Hep B ☐ Others ☐ (specify)____

E. POSTPARTUM ASSESSMENT OF THE MOTHER (Tick a, b or c. Tick all complications observed)

a. Woman died ☐ → Complete Outcomes Sheet and other documentation
b. Woman healthy ☐ → Go to the next Section of this Case Sheet
c. Woman with complications ☐
   Pregnancy induced hypertension ☐ Pre-eclampsia ☐ Eclampsia ☐ → Manage and refer using Complication Sheet B
   Infection with or without shock ☐ → Manage and refer using Complication Sheet D
   Postpartum haemorrhage ☐ → Manage and refer using Complication Sheet F
   Other ☐ (specify)____________________________________ → Go to Referral Sheet H

F. POSTPARTUM ASSESSMENT OF THE NEWBORN (Tick a, b or c. Tick all complications observed)

a. Newborn died ☐ → Complete Outcomes Sheet and other documentation
b. Newborn healthy ☐ → Go to the next Section of this Case Sheet
c. Newborn with complications ☐
   Infection / Sepsis ☐ } → Manage and refer using Complication Sheet G
   Other ☐ (specify) ____________________________________ }

Date diagnosis made [| | | | |] Time diagnosis made [|] hrs [|] mins

Diagnosed by SN ☐ MO ☐ Other ☐ Name of the staff ____________________________ Signature ____________________________

OUTCOMES SHEET

Fill this sheet for women and/or newborns discharged together without either being referred.

Fill this sheet also if either or both of them die at the PHC

A.BACKGROUND INFORMATION

District ______________________ Taluka ____________________ PHC Location __________________

Name ________________________ Thayi card number ☐☐☐☐☐☐☐☐

Address ____

Contact number ☐☐☐☐☐☐☐☐☐☐

Date of admission ☐☐☐☐☐☐☐☐ Time of admission ☐☐ hrs ☐☐ mins

Date of delivery ☐☐☐☐☐☐☐☐ Time of delivery ☐☐ hrs ☐☐ mins

Date of discharge / death ☐☐☐☐☐☐☐☐ Time of discharge / death ☐☐ hrs ☐☐ mins

B. OUTCOMES

Maternal outcome (Tick either a, b, c or d)

a. Woman healthy with no complications ☐

b. Woman healthy after being treated for complications at the PHC ☐ Specify ______________________

c. Woman died ☐

During initial assessment ☐ During labour ☐ During delivery ☐ During postpartum period ☐

Possible cause of death ___________________________

d. Woman referred for complication (if newborn died) ☐ Specify complication ______________________

Newborn outcome (Tick either a, b, c or d)

a. Live birth ☐

Newborn healthy with no complications ☐

Newborn healthy after being treated for complications at the PHC ☐ Specify ______________________

b. Newborn referred for complication (if mother died) ☐ Specify complication ______________________

c. Still birth ☐ Fresh ☐ Macerated ☐

d. Newborn died ☐

During delivery ☐ During postpartum period ☐

Possible cause of death ___________________________

C. DELIVERY DETAILS

Mode of delivery Spontaneous cephalic ☐ Instrumental ☐ Breech ☐

Maturity Preterm (<37 wks) ☐ Term (37 to 42 wks) ☐ Post term (>42 wks) ☐ Sex Male ☐ Female ☐

Weight ☐☐☐☐ gms Breastfeeding initiated Time ☐☐ hrs ☐☐ mins

Newborn Immunization and medications provided BCG ☐ 0 OPV ☐ 0 Hepatitis B ☐ Vit K ☐

D. FOLLOW UP IN THE COMMUNITY

Informed ANM and ASHA Y ☐ N ☐

Name of the ANM________________________________Telephone number_________________________

Name of the ASHA_______________________________ Telephone number_________________________

Follow up instructions for ANM/ ASHA Y ☐ N ☐ If Yes, Specify

____

____

____

Date of follow up at PHC ______________________

Name of the staff__________________________ Signature ___________________________

COMPLICATION CASE SHEET A

For initial management and referral for pregnant women with prolonged/ obstructed labour/ rupture of membranes >12 hours

Send a copy of the case sheet along with this sheet to the referral facility

A.BACKGROUND INFORMATION

District ______________________ Taluka __________________ PHC Location _________________

Name_______________________________ Thayi card number | | | | | | | | |

Address ____

B.SPECIFIC DIAGNOSIS AND INITIAL MANAGEMENT

Diagnosis (Tick a, b or c)

a. ☐ Prolonged labour (Any one of the following must be present. Tick boxes as appropriate)

- ☐ Plotted cervical dilatation line in the partograph is to the right of Line A at the four hour and eight hour assessments
- ☐ Contractions do not increase in frequency and duration
- ☐ Cervix not dilated beyond 4 cm after 8 hrs of regular contractions
- ☐ Cervix not dilating at least 1 cm an hour in active labour (regular contractions and initial PV of 3-4 cm)
- ☐ No cervical change with repeat PV after 4 hours in active phase of labour
- ☐ Full dilation of cervix but no descent of foetal head despite maternal pushing efforts
- ☐ Two contractions or less in 10 minutes lasting less than 40 seconds

b. ☐ Obstructed labour (Any two of the following must be present. Tick boxes as appropriate)

- ☐ Plotted cervical dilatation line in the partograph is to the right of Line A at the four hour and eight hour assessments
- ☐ No cervical change (secondary arrest) with repeat PV after 4 hours in active phase of labour
- ☐ Significant caput and moulding
- ☐ Cervix that is not well applied to presenting part
- ☐ Swollen, oedematous cervix
- ☐ Ballooning lower uterine segment
- ☐ Formation of retraction band felt over abdomen
- ☐ Foetal or maternal distress
- ☐ Labour that is longer than 24 hours duration

c. ☐ Woman has rupture of membranes for greater than 12 hours after 37 weeks of pregnancy with or without contractions

Initial management at the PHC (Tick when management done)

THESE ARE OBSTETRIC EMERGENCIES

- ☐ Call and determine the nearest facility where a caesarean section can be done if necessary
- ☐ Arrange transport
- ☐ Keep the woman nil per orally (NPO)
- ☐ Do NOT give Oxytocin
- ☐ Insert 16-18 gauge IV and give IV Normal Saline or Ringer's Lactate or 5% Dextrose Normal Saline at 30 drops per min
- ☐ Insert Foley's catheter in case of prolonged/obstructed labour

Reason if not given_________________________

Give the following antibiotics

- ☐ Ampicillin 1g Orally ☐ or IV ☐ and
- ☐ Metronidazole 400mg Orally ☐ or 500mg IV ☐ and
- ☐ Gentamicin 80mg IM ☐ or IV ☐

Reason if not given_________________________

Any other antibiotics given (specify)______________

____

If gestational age is between 24 and 34 weeks

- ☐ Give corticosteroids either Betamethasone IM 12mg ☐ or Dexamethasone IM 6 mg ☐

Reason if not given_________________________

Date | | | | | Time | | | | |

Arrange for the following during transportation

- ☐ Keep the woman in left lateral position
- ☐ Continue fluid and carry extra bottles to last till she reaches the facility
- ☐ Give oxygen if foetal distress is present
- ☐ Keep a delivery set and essential drugs handy
- ☐ SN accompanies the woman and monitors fluid intake and urine output

C. CONDITION AT THE TIME OF REFERRAL

Consciousness Conscious ☐ Semiconscious ☐ Unconscious ☐ Pulse ________/min BP ____________mmHg

Temperature _____ ^{0}C Respiration _______/min FHR ________/ min other (specify) ____________________________

D.REFERRAL DETAILS

Date diagnosis made | | | | | | | Time diagnosis made | | | hrs | | | mins

Diagnosed by SN ☐ MO ☐ Other ☐ Staff name _________________________ Signature ______________________

Details of facility to which the woman is referred Name __________________Location ________________District ____________________Taluka ______________________ Type CHC ☐ TH ☐ DH ☐ Private ☐ Other ☐ (specify)______________

Person to contact __________________________Telephone # ____

Date left PHC | | | | | | | Time left PHC | | | | | |

Type of vehicle used for transportation 108 ☐ Ambulance Govt. ☐ Ambulance Pvt ☐ Other specify ☐_________________

Outcome of referral Discharged healthy ☐ Died ☐ Date | | | | | | | Time | | | hrs | | | mins

Project Sukshema | Department of Health, Government of Karnataka | National Rural Health Mission

COMPLICATION CASE SHEET B

For initial management and referral for women with pregnancy induced hypertension/ pre-eclampsia/ eclampsia (pregnant, in labour or postpartum). Send a copy of the case sheet along with this sheet to the referral facility

A.BACKGROUND INFORMATION	
District ______________________ Taluka ____________________ PHC Location ____________________	
Name______________________________ Thayi card number ☐☐☐☐☐☐☐☐	
Address ____	
B.SPECIFIC DIAGNOSIS AND INITIAL MANAGEMENT	
Diagnosis (Tick a, b, c, d or e)	Initial management at the PHC (Tick when management done)
a. ☐ Mild PIH (Tick boxes; all three must apply)<br>☐ BP ≥140/90 and ≤160/110<br>☐ No proteinuria<br>☐ Gestation ≥ 20 weeks<br><br>b. ☐ Mild pre-eclampsia (Tick boxes; all three must be present)<br>☐ BP ≥140/90 and ≤160/110<br>☐ Proteinuria<br>☐ Gestation ≥ 20 weeks	☐ If either in labour or not in labour, call and determine the nearest facility where further laboratory assessment can be done and where labour induction, augmentation of labour and caesarean section could be done if necessary<br>☐ If the delivery is imminent, conduct the delivery<br>☐ If postpartum, call and determine the nearest facility that can do further laboratory assessment and management<br>☐ Arrange transport
c. ☐ Severe PIH (Tick boxes; all three must apply)<br>☐ BP ≥160/110<br>☐ No proteinuria<br>☐ Gestation ≥ 20 weeks	☐ If either in labour or not in labour, call and determine the nearest facility where labour induction, augmentation of labour and caesarean section could be done if necessary<br>☐ If the delivery is imminent, conduct the delivery and refer to the same facility for observation and care<br>☐ If postpartum, call and determine the nearest facility that can do further laboratory assessment and management<br>☐ Arrange transport<br>Give the following<br>☐ Antihypertensive, either Hydralazine 5mg IV ☐ or Nifedipine 5mg Orally ☐<br>Reason if not given______________________<br>☐ Start IV fluids Ringer's Lactate or Normal Saline or 5% Dextrose Normal Saline at 30 drops per min<br>Reason if not given______________________<br>If gestational age is between 24 and 34 weeks<br>☐ Give corticosteroids either Betamethasone IM 12mg ☐ or Dexamethasone IM 6 mg ☐<br>Reason if not given______________________
d. ☐ Severe pre-eclampsia (Tick boxes; all three must be present)<br>☐ BP ≥160/110<br>☐ Proteinuria<br>☐ Gestation ≥ 20 weeks	THIS IS AN OBSTETRIC EMERGENCY<br>☐ If either in labour or not in labour, call and determine the nearest facility where labour induction, labour augmentation and caesarean section could be done if necessary<br>☐ If the delivery is imminent, conduct the delivery and refer to the same facility for observation and care<br>☐ If postpartum, call and determine the nearest facility that can do further laboratory assessment and management<br>☐ Arrange transport<br>Give the following<br>☐ Oxygen<br>☐ Antihypertensive, either Hydralazine 5mg IV ☐ or Nifedipine 5mg Orally ☐<br>Reason if not given______________________<br>☐ Magnesium Sulphate (preferred)10ml (5gm) IM in each buttock total 20ml (10gm) **or**<br>☐ Diazepam 20mg (4ml) Rectally in 10ml syringe ☐ or 10mg IV slowly over 2 mins ☐<br>Reason if not given______________________<br>☐ Start IV fluids Ringer's Lactate or Normal Saline or 5% Dextrose Normal Saline at 60 drops per min

	☐ Insert Foley's catheter<br>After giving Magnesium Sulphate, if respiratory rate decreases to <16/min or if patellar deep tendon reflexes are absent,<br>☐ Give Calcium Gluconate 1g IV over 10 mins<br>If gestational age is between 24 and 34 weeks<br>☐ Give corticosteroids either Betamethasone IM 12mg ☐ or Dexamethasone IM 6 mg ☐<br>Reason if not given_______________________
e. ☐ **Eclampsia** (First three must be present. Tick the fourth as appropriate)<br>☐ BP ≥140/90<br>☐ Fits/convulsions<br>☐ Gestation ≥ 20 weeks up to 6 weeks postpartum<br>☐ Proteinuria	<u>THIS IS AN OBSTETRIC EMERGENCY</u><br>☐ If either in labour or not in labour, call and determine the nearest facility where further laboratory assessment can be done and where labour induction, augmentation of labour and caesarean section could be done if necessary or<br>☐ If postpartum, call and determine the nearest facility that can do further laboratory assessment and management<br>☐ If the delivery is imminent, conduct the delivery and refer to the same facility for observation and care<br>☐ Arrange transport<br>☐ Clean mouth and nose, and put mouth gag. Do NOT restrain<br>Give following<br>☐ Oxygen<br>☐ Magnesium Sulphate (preferred) 10ml (5gm) IM in each buttock total 20ml (10gm) or<br>☐ Diazepam 20mg (4ml) Rectally in 10ml syringe ☐ or 10mg IV slowly over 2 mins☐<br>Reason if not given_______________________<br>☐ Start IV fluids Ringer's Lactate or Normal Saline or 5% Dextrose Normal Saline slowly<br>☐ Insert Foley's catheter<br>Reason if not given_______________________<br>If diastolic BP >110 mmHg,<br>☐ Give antihypertensive, either Hydralazine 5mg IV ☐ or Nifedipine 5mg Orally ☐<br>Reason if not given_______________________<br>After giving Magnesium Sulphate, if respiratory rate decreases to <16/min or if patellar deep tendon reflexes are absent,<br>☐ Give Calcium Gluconate 1g IV over 10 mins<br>If gestational age is between 24 and 34 weeks<br>☐ Give corticosteroids either Betamethasone IM 12mg ☐ or Dexamethasone IM 6 mg ☐<br>Reason if not given_______________________

Date of management ☐☐☐☐☐☐ Time of management ☐☐ hrs ☐☐ mins

Arrange for the following during transportation

☐ Keep the woman in left lateral position
☐ Continue fluid and carry extra bottles to last till she reaches the facility
☐ Give oxygen (if required)
☐ Keep a delivery set and essential drugs handy
☐ SN accompanies the woman and monitors fluid intake and urine output
☐ Ensure baby is kept warm, feedings continued if the woman is postpartum

C. CONDITION AT THE TIME OF REFERRAL

Consciousness Conscious ☐ Semiconscious ☐ Unconscious ☐ Pulse ________/min BP ____________mmHg

Temperature _____ ^{0}C Respiration _______/min FHR ______/ min Patellar deep tendon reflex Present ☐ Absent ☐

Urine output ______cc/hr Other specify ______________________________

D.REFERRAL DETAILS

Date diagnosis made ☐☐☐☐☐☐ Time diagnosis made ☐☐ hrs ☐☐ mins

Diagnosed by SN ☐ MO ☐ Other ☐ Staff name ____________________ Signature ____________________

Details of facility to which the woman is referred

Name ________________Location ______________District __________________Taluka ____________________

Type CHC ☐ TH ☐ DH ☐ Private ☐ Other ☐ (specify)________________________

Person to contact ______________________Telephone # ______________________________________

Date left PHC ☐☐☐☐☐☐ Time left PHC ☐☐ ☐☐

Type of vehicle used for transportation 108 ☐ Ambulance Govt. ☐ Ambulance Pvt ☐ Other specify ☐________________

Outcome of referral Discharged healthy ☐ Died ☐ Date ☐☐☐☐☐☐ Time ☐☐ hrs ☐☐ mins

Project Sukshema Department of Health, Government of Karnataka National Rural Health Mission

COMPLICATION CASE SHEET C

For initial management and referral for pregnant women with antepartum haemorrhage

Send a copy of the case sheet along with this sheet to the referral facility

A.BACKGROUND INFORMATION

District ____________________________ Taluka _______________________ PHC Location _____________________

Name_____________________________________ Thayi card number | | | | | | | | |

Address ____

B.SPECIFIC DIAGNOSIS AND INITIAL MANAGEMENT

Diagnosis (Tick a, b or c)

a. □ **Placental abruption** (First two must be present. Tick third as appropriate)
- □ Abdominal pain (if contractions are present and the pain is present in between contractions)
- □ Tense or tender uterus on palpation
- □ Bleeding from the vagina (if concealed there may be no bleeding seen)

b. □ **Placenta praevia** (Tick boxes; all must apply)
- □ No abdominal pain (the woman is pain free or if having contractions has no pain between contractions)
- □ Bleeding from the vagina now or prior
- □ Relaxed uterus on palpation / uterus irritable

c. □ **Ruptured uterus** (Tick boxes; all must be present)
- □ History of severe abdominal pain that may have suddenly decreased
- □ Bleeding from vagina or into abdomen
- □ Abnormal uterine contour on exam (Bandl's ring)
- □ Easily palpable foetal parts

Initial management at the PHC (Tick when management done)

<u>THESE ARE OBSTETRIC EMERGENCIES – DO NOT PERFORM VAGINAL EXAM</u>

- □ Call and determine the nearest facility where a caesarean section can be done if necessary and the required blood type is available
- □ Arrange transport □ Arrange for a blood donor □ Give oxygen
- □ Insert Foley's catheter □ Keep the woman NPO □ Do NOT give Oxytocin

Record vital signs Pulse ___/min BP ________mmHg Respiration ____/min Temp ____ ^{0}C

If the woman is in shock (systolic BP <90 mmHg, and/or pulse >110/minute) or she is bleeding heavily (soaking one pad in less than 5 minutes)

- □ Insert 16-18 gauge IV and give IV Normal Saline or Ringer's Lactate or 5 % Dextrose Normal Saline at 60 drops per min for the first 1 litre (2 bottles) □ Next 500ml (1 bottle) at 30 drops per min □ Repeat if necessary

If the systolic BP increases to ≥100 mmHg and pulse slows down to <100/min,

- □ Slow the IV drip to 3 drops per min □ Keep the woman warm, keep her feet elevated

If the woman is not in shock or she is not bleeding heavily

- □ Insert 16-18 gauge IV and give IV Normal Saline or Ringer's Lactate or 5% Dextrose Normal Saline at 30 drops per min 500ml (1 bottle) □ Repeat if necessary

If ruptured uterus is suspected, give the following antibiotics

- □ Ampicillin 1gm IV (preferred) □ or 500mg Orally □ and
- □ Metronidazole 500mg IV (preferred) □ or 400mg Orally □ and
- □ Gentamicin 80mg IM □ or IV □
- □ Any other antibiotics given (specify)________________________________

Reason if not given____________________________________

If gestational age is between 24 and 34 weeks

- □ Give corticosteroids either Betamethasone IM 12mg □ or Dexamethasone IM 6 mg □

Reason if not given____________________________

Date | | | | | Time | | | | |

Arrange for the following during transportation

- □ Keep the woman's legs elevated □ Continue to keep the woman warm
- □ Continue fluid and carry extra bottles to last till she reaches the facility
- □ Give oxygen if foetal distress is present □ Keep a delivery set and essential drugs handy
- □ SN accompanies the woman to monitors fluid intake, urine output and other vitals every 10mins

C. CONDITION AT THE TIME OF REFERRAL

Consciousness Conscious □ Semiconscious □ Unconscious □ Pulse _____/min BP ___________mmHg Temperature ___ ^{0}C

Respiration _______/min FHR ______ / min Urine output ______cc/hr Other specify ____________________________

D.REFERRAL DETAILS

Date diagnosis made | | | | | | | Time diagnosis made | | | hrs | | | mins

Diagnosed by SN □ MO □ Other □ Staff name _________________________ Signature _______________________

Details of facility to which the woman is referred

Name ________________Location ____________________District __________________________Taluka ___________________

Type CHC □ TH □ DH □ Private □ Other □ (specify)____________________________

Person to contact __________________________Telephone # ____

Date left PHC | | | | | | | Time left PHC | | | hrs | | | mins

Type of vehicle used for transportation 108 □ Ambulance Govt □ Ambulance Pvt □ Other specify □ ______________________

Outcome of referral Discharged healthy □ Died □ Date | | | | | | | Time | | | hrs | | | mins

COMPLICATION CASE SHEET D

For initial management and referral for women with infection/sepsis (pregnant or postpartum)

Send a copy of the case sheet along with this sheet to the referral facility

A.BACKGROUND INFORMATION

District ______________________________ Taluka ________________________ PHC Location ________________________

Name____ Thayi card number | | | | | | | | |

Address ____

B.SPECIFIC DIAGNOSIS AND INITIAL MANAGEMENT

Record vital signs Pulse ___/min BP _________mmHg Respiration ____breaths/min Temperature ____ ^{0}C

If the woman is in septic shock (systolic BP <90 mmHg, and/or pulse >110/minute) give the following

☐ Insert 16-18 gauge IV and give IV Normal Saline or Ringer's Lactate or 5% Dextrose Normal Saline at 60 drops per min for the first 1 litre (2 bottles) ☐ Next 500ml (1 bottle) at 30 drops per min ☐ Repeat if necessary

☐ **If the systolic BP increases to ≥100** mmHg and pulse slows down to <100/min, slow the IV drip to 3 drops per min

☐ Give the following antibiotics Ampicillin 2g IV ☐ and Metronidazole 400mg Orally ☐ or 500mg IV ☐ and Gentamicin 80mg IM ☐ or IV ☐

☐ Keep the woman warm, keep her feet elevated ☐ Give oxygen ☐ Insert Foley's catheter ☐ Keep the woman NPO

Diagnosis (Tick a, b, c, d, e or f)	Initial management at the PHC (Tick when management done. If in septic shock and antibiotics are already administered, do not repeat the antibiotics)
a. ☐ Acute pyelonephritis (Tick boxes, all must be present)<br>☐ Burning sensation while passing urine<br>☐ Flank pain<br>☐ Fever (temperature ≥ 38 ^{0}C) and chills	☐ Call and determine the nearest facility with intensive care unit (ICU) for admission and treatment<br>☐ Arrange transport<br>Give the following antibiotics<br>☐ Ampicillin 2g IV and<br>☐ Gentamicin 80mg IM ☐ or IV ☐<br>Reason if not given ________________________________<br>☐ IV Normal Saline or Ringer's Lactate or 5% Dextrose Normal Saline at 30 drops per min<br>☐ Paracetamol 500mg Orally
b. ☐ Amnionitis in pregnancy (First 3 must be present. Tick the 4th as appropriate)<br>☐ Fever (temperature ≥ 38 ^{0}C)<br>☐ Rupture of membranes<br>☐ Abdominal pain<br>☐ Foul smelling or purulent discharge per vagina	☐ Call and determine the nearest facility where labour induction, labour augmentation, caesarean section and ICU are available if needed<br>☐ Arrange transport<br>☐ If the delivery is imminent, conduct the delivery<br>Give the following antibiotics (If the condition is poor, IV is preferred)<br>☐ Ampicillin 1gm Orally ☐ or IV ☐ and<br>☐ Metronidazole 400mg Orally ☐ or 500mg IV ☐ and<br>☐ Gentamicin 80mg IM ☐ or IV ☐<br>Reason if not given________________________________<br>☐ Paracetamol 500mg Orally
c. ☐ Endometritis/ Puerperal sepsis in postpartum (First 4 must be present. Tick the 5th as appropriate)<br>☐ Fever (temperature ≥ 38 ^{0}C)<br>☐ Abdominal pain<br>☐ Tender uterus<br>☐ Foul smelling or purulent lochia<br>☐ Increased vaginal bleeding	☐ Call and determine the nearest facility where dilatation and curettage (D&C) and intensive care unit (ICU) are available<br>☐ Arrange transport<br>☐ If the delivery is imminent, conduct the delivery<br>Give the following antibiotics (If the condition is poor, IV is preferred)<br>☐ Ampicillin 1g Orally ☐ or IV ☐ and<br>☐ Metronidazole 400mg Orally ☐ or 500mg IV ☐ and<br>☐ Gentamicin 80mg IM ☐ or IV ☐<br>Reason if not given________________________________<br>☐ Paracetamol 500mg Orally<br>☐ If bleeding heavily, give either Oxytocin 10 IU IM ☐ or IV ☐ or Misoprostol 600 mcg Rectally ☐ or Orally ☐ or Methergine 0.2 mg IM ☐ or IV ☐
d. ☐ Mastitis (Both must be present)<br>☐ Red and tender breast<br>☐ Fever (temperature ≥ 38 ^{0}C)	☐ Give warm compress on the breasts before feeding<br>☐ Encourage continuous breastfeeding and emptying of breasts<br>Give the following antibiotics, either<br>☐ Cloxacillin 500 mg Orally four times daily for 10 days or<br>☐ Erythromycin 250 mg Orally three times daily for 10 days or

	☐ Ampicillin 500mg Orally three times daily for 10 days<br>Reason if not given________________<br>☐ Review after 3 days, if no improvement, change the antibiotic<br>☐ If there is improvement, continue for another 7 days.<br>☐ Refer if abscess develops or if there is no improvement despite changing the antibiotic
e. ☐ Breast abscess (First 3 must be present. Tick the 4th as appropriate)<br>☐ Tender, discreet fluctuant mass in the breast<br>☐ Fever (temperature ≥ 38 ^{0} C) and chills<br>☐ Pain in the breast<br>☐ Pus draining	☐ Call and determine the nearest facility where breast abscess can be surgically drained, if necessary<br>☐ Arrange transport<br>☐ Apply warm compress, give breast support, encourage mother to breastfeed<br>Give the following antibiotics, either<br>☐ Cloxacillin 500 mg Orally or<br>☐ Ampicillin 500mg Orally or<br>☐ Erythromycin 250mg Orally<br>Reason if not given________________<br>☐ Paracetamol 500mg Orally
f. ☐ Wound infection / abscess (First 2 must be present. Tick the 3rd as appropriate)<br>☐ Fever (temperature ≥ 38^0 C)<br>☐ Red, swollen, painful wound<br>☐ Pus draining	If the wound infection is mild (no abscess), give the following antibiotics<br>☐ Ampicillin 500mg four times daily Orally and<br>☐ Metronidazole 400mg three times daily Orally<br>☐ Have the woman return after one day for reassessment<br>☐ If there is improvement after the 1st day, continue for another 4 days<br>☐ Refer if there is no improvement after 1st day<br><br>If there is wound abscess<br>☐ Call and determine the nearest facility where abscess can be surgically drained<br>☐ Arrange transport<br>Give the following antibiotics<br>☐ Ampicillin 1g Orally ☐ or IV ☐ and<br>☐ Metronidazole 400mg Orally ☐ or 500mg IV ☐ and<br>☐ Gentamicin 80mg IM ☐ or IV ☐<br>Reason if not given________________<br>☐ Paracetamol 500mg Orally

Date of management ☐☐☐☐☐☐ Time of management ☐☐ hrs ☐☐ mins

Arrange for the following during transportation

- ☐ In case of shock
  - ☐ Continue fluids and carry extra bottles to last till she reaches the facility
  - ☐ Give oxygen (if required)
  - ☐ SN accompanies the woman and monitors fluid intake and urine output and other vitals every 10 mins
- ☐ If the woman is pregnant, keep a delivery set and essential drugs handy
- ☐ If delivered, ensure baby is kept warm, feedings continued

C. CONDITION AT THE TIME OF REFERRAL

Consciousness: Conscious ☐ Semiconscious ☐ Unconscious ☐ Pulse: ________/min BP: ____________mmHg
Temperature: _____^{0} C Respiration: _______/min Other specify________________

D.REFERRAL DETAILS

Date diagnosis made ☐☐☐☐☐☐ Time diagnosis made ☐☐ hrs ☐☐ mins

Diagnosed by SN ☐ MO ☐ Other ☐ Staff name ________________ Signature ________________

Details of facility to which the woman is referred

Name ________________Location ________________District ________________Taluka ________________

Type CHC ☐ TH ☐ DH ☐ Private ☐ Other ☐ (specify)________________

Person to contact ________________Telephone # ________________

Date left PHC ☐☐☐☐☐☐ Time left PHC ☐☐ hrs ☐☐ mins

Type of vehicle used for transportation 108 ☐ Ambulance Govt ☐ Ambulance Pvt ☐ Other specify ☐ ________________

Outcome of referral Discharged healthy ☐ Died ☐ Date ☐☐☐☐☐☐ Time ☐☐ hrs ☐☐ mins

COMPLICATION CASE SHEET E

For initial management and referral for women with preterm labour with or without rupture of membranes / premature rupture of membranes with or without preterm labour. Send a copy of the case sheet along with this sheet to the referral facility

A.BACKGROUND INFORMATION

District ______________________________ Taluka ________________________ PHC Location _______________________

Name____ Thayi card number ☐☐☐☐☐☐☐☐

Address ____

B.SPECIFIC DIAGNOSIS AND INITIAL MANAGEMENT

Diagnosis (Tick a, b, c or d)	Initial management at the PHC (Tick when management done)
a. ☐ **Preterm labour with or without rupture of membranes and with fever** (The first three must apply. Tick fourth as appropriate)<br>☐ Gestational age between 24 and 37 weeks<br>☐ Active labour<br>☐ Temp ≥38 C<br>☐ Rupture of membranes	☐ **Call and determine the facility for preterm delivery and special newborn care**<br>☐ **Arrange transport**<br>**Give the following antibiotics**<br>☐ Ampicillin 1g Orally ☐ or IV ☐ and<br>☐ Metronidazole 400mg Orally ☐ or 500mg IV ☐ and<br>☐ Gentamicin 80mg IM ☐ or IV ☐<br>Reason if not given ________________________________<br>**If gestational age is between 24 and 34 weeks**<br>☐ Give corticosteroids either Betamethasone IM 12mg ☐ or Dexamethasone IM 6 mg ☐<br>Reason if not given ________________________________
b. ☐ **Preterm labour with or without rupture of membranes and no fever** (The first three must apply. Tick fourth as appropriate)<br>☐ Gestational age between 24 and 37 weeks<br>☐ Active labour<br>☐ No fever<br>☐ Rupture of membranes	☐ **Call and determine the facility for preterm delivery and special newborn care**<br>☐ **Arrange transport**<br>**Give the following antibiotic**<br>☐ Ampicillin 1g Orally ☐ or IV ☐<br>Reason if not given________________________________<br>**If gestational age is between 24 and 34 weeks**<br>☐ Give corticosteroids either Betamethasone IM 12mg ☐ or Dexamethasone IM 6 mg ☐<br>Reason if not given________________________________
c. ☐ **Preterm rupture of membranes without labour and without fever** (Tick boxes; all must apply)<br>☐ Gestational age between 24 and 37 weeks<br>☐ Rupture of membranes<br>☐ Not in active labour<br>☐ No fever	☐ **Call and determine the facility for labour induction, preterm delivery and special newborn care**<br>☐ **Arrange transport**<br>**Give the following antibiotics**<br>☐ Erythromycin 250 mg Orally and<br>☐ Amoxicillin 500 mg Orally<br>Reason if not given ________________________________<br>**If gestational age is between 24 and 34 weeks**<br>☐ Give corticosteroids either Betamethasone IM 12mg ☐ or Dexamethasone IM 6 mg ☐<br>Reason if not given ________________________________
d. ☐ **Preterm rupture of membranes without labour and with fever** (Tick boxes; all must apply)<br>☐ Gestational age between 24 and 37 weeks<br>☐ Rupture of membranes<br>☐ Not in active labour<br>☐ Temp ≥ 38 C	☐ **Call and determine the facility for labour induction, preterm delivery and special newborn care**<br>☐ **Arrange transport**<br>**Give the following antibiotics**<br>☐ Ampicillin 1g Orally ☐ or IV ☐ and<br>☐ Metronidazole 400mg Orally ☐ or 500mg IV ☐ and<br>☐ Gentamicin 80mg IM ☐ or IV ☐<br>Reason if not given ________________________________<br>**If gestational age is between 24 and 34 weeks**<br>☐ Give corticosteroids either Betamethasone IM 12mg ☐ or Dexamethasone IM 6 mg ☐<br>Reason if not given ________________________________

Date of management ☐☐☐☐☐☐ Time of management ☐☐ hrs ☐☐ mins

Arrange for the following during transportation

☐ Give oxygen

☐ Keep the woman in left lateral position

C. CONDITION AT THE TIME OF REFERRAL

Consciousness: Conscious ☐ Semiconscious ☐ Unconscious ☐ Pulse: ________/min BP: _____________mmHg
Temperature: _____ ^{0}C Respiration: _______/min Other specify ______________________________________

D.REFERRAL DETAILS

Date diagnosis made ☐☐☐☐☐☐ Time diagnosis made ☐☐ hrs ☐☐ mins

Diagnosed by SN ☐ MO ☐ Other ☐ Staff name ___________________________ Signature ________________________
Details of facility to which the woman is referred
Name ________________Location _____________________District ___________________________Taluka ____________________

Type CHC ☐ TH ☐ DH ☐ Private ☐ Other ☐ (specify)______________________________
Person to contact ____________________________Telephone # ____
Date left PHC ☐☐☐☐☐☐ Time left PHC ☐☐ hrs ☐☐ mins

Type of vehicle used for transportation 108 ☐ Ambulance Govt ☐ Ambulance Pvt ☐ Other specify ☐ ________________________

Outcome of referral Discharged healthy ☐ Died ☐ Date ☐☐☐☐☐☐ Time ☐☐ hrs ☐☐ mins

COMPLICATION CASE SHEET F

For initial management and referral for women with postpartum haemorrhage

Send a copy of the case sheet along with this sheet to the referral facility

A.BACKGROUND INFORMATION

District ______________________________ Taluka _________________________ PHC Location _______________________

Name____ Thayi card number | | | | | | | | |

Address ____

B.SPECIFIC DIAGNOSIS AND INITIAL MANAGEMENT

☐ Shout for help. Mobilize all available health personnel

Record vital signs **Pulse** ___/min BP _________mmHg **Respiration** ____breaths/min **Temperature** ____ ^{0} C

☐ Insert 16-18 gauge IV and give Normal Saline or Ringer's Lactate or 5 % Dextrose Normal Saline at 30 drops per min

☐ Insert Foley's catheter

If the woman is in shock (systolic BP <90 mmHg, and/or pulse >110/minute) or she is bleeding heavily (soaking one pad in less than 5 minutes) give the following

- ☐ Give IV Normal Saline or Ringer's Lactate or 5 % Dextrose Normal Saline **at 60 drops per min** for the first 1 litre (2 bottles)
- ☐ Next 500ml (1 bottle) at 30 drops per min ☐ Repeat if necessary
- ☐ **If the systolic BP increases to ≥100** mmHg **and pulse slows down to** <100/min, slow the IV drip to 3 drops per min
- ☐ Keep the woman warm, keep her feet elevated ☐ Give oxygen ☐ Keep the woman NPO

Diagnosis (Tick a, b, c, or d)	Initial management at the PHC (Tick when management done)
a. ☐ **Retained placenta/ fragments within first 24 hours postpartum** (Tick boxes; both must be present)<br>☐ Increased bleeding<br>☐ Placenta not delivered either completely or partially	☐ **Give Oxytocin 20 IU in IV infusion in 500 ml of Ringer's Lactate or Dextrose Normal Saline at 60 drops per minute**<br>**Give the following antibiotics (prophylaxis prior to performing manual removal)**<br>☐ Ampicillin 1g IV ☐ or Orally ☐ and<br>☐ Metronidazole 500mg IV ☐ or 400 mg Orally ☐ and<br>☐ Gentamicin 80mg IV ☐ or IM ☐<br>Reason if not given ___________________________________<br><br>☐ **Perform manual removal of placenta or fragments**<br>Reason if not done ___________________________________<br>**If unable to perform manual removal**<br>☐ **Call and determine the nearest facility where blood and surgical intervention is available, if necessary**<br>☐ **Arrange transport**<br>☐ **Continue Oxytocin 20 IU in 500ml of Ringer's Lactate or Normal Saline or 5 % Dextrose Normal Saline at 30 drops per min**<br>☐ **Perform bimanual compression of the uterus if bleeding is heavy during transport**
b. ☐ **Atonic uterus** (Tick boxes; all must be present)<br>☐ Increased bleeding<br>☐ Placenta expelled<br>☐ Soft and flabby uterus	☐ **Massage the uterus to expel any clots**<br>☐ **If not given earlier, give Oxytocin 10 IU IM**<br>☐ **Give Oxytocin 20 IU in IV infusion in 500 ml of Ringer's Lactate or Normal Saline or 5% Dextrose Normal Saline at 60 drops per minute**<br>☐ **Follow up with Oxytocin 10 IU in IV infusion in 500 ml of Ringer's Lactate or Normal Saline or 5% Dextrose Normal Saline at 40 drops per minute**<br>Reason if not given ___________________________________<br><br>**If bleeding continues, give one of the following additional uterotonics, either**<br>☐ Methergine/ Ergotamine 0.2 mg IM per dose ☐ can be repeated every 15 minutes for a total of 5 doses. **Do NOT give if high blood pressure is present.** Repeat doses given ☐ Number of doses:________ or<br>☐ Prostaglandin/ Carboprost 0.25 mg IM per dose ☐ can be repeated every 15 minutes for a total of 8 doses. **Do NOT give if patient has asthma.** Repeat doses given ☐ Number of doses:_____ or<br>☐ Misoprostol 600 mcg Orally ☐ or Rectally ☐<br>Reason if not given ___________________________________

	If bleeding continues and uterus is flabby<br>☐ Perform bimanual compression of uterus<br>☐ Call and determine the nearest facility where blood and surgical intervention is available, if necessary<br>☐ Arrange transport<br>☐ Continue Oxytocin 20 IU in 500ml of Ringer's Lactate or Dextrose Normal Saline at 30 drops per min
c. ☐ Perineal/ cervical tears (Tick boxes; all must be present)<br>☐ Increased bleeding<br>☐ Placenta expelled<br>☐ Uterus well contracted	☐ Suture, if easily accessible (first and second degree tears)<br><br>If not easily accessible or identified (or third and fourth degree tears)<br>☐ Pack the vagina with sterile dressing<br>Give the following prophylactic antibiotic in case of fourth degree tear<br>☐ Ampicillin 500 mg Orally<br>☐ Metronidazole 400 mg Orally<br>Reason if not given ______________________<br>☐ Call and determine the facility for surgical intervention, if necessary<br>☐ Arrange transport
d. ☐ Delayed PPH due to retained placental fragments (Tick the box)<br>☐ Bleeding 24 hours after delivery not due to any other cause	☐ Call and determine the nearest facility for dilatation and curettage (D&C), if necessary<br>☐ Arrange transport<br>☐ Give either Oxytocin 10 IU IM ☐ or IV ☐ or Misoprostol 600 mcg Rectally ☐ or Orally ☐<br>If there is foul smelling discharge, give the following antibiotics<br>☐ Ampicillin 1 gm Orally ☐ or IV ☐ and<br>☐ Metronidazole 400 mg Orally ☐ or 500 mg IV ☐ and<br>☐ Gentamicin 80 mg IM ☐ or IV ☐<br>Reason if not given ____

Date of management ☐☐☐☐☐☐ Time of management ☐☐ hrs ☐☐ mins

Arrange for the following during transportation

- ☐ In case of shock
  - ☐ Continue fluids and carry extra bottles to last till she reaches the facility
  - ☐ Give oxygen (if required)
  - ☐ Keep the woman warm and raise her legs
  - ☐ SN accompanies the woman and monitors fluid intake, urine output and other vital signs every 10 mins
- ☐ Call and arrange for blood / donor, if necessary
- ☐ Ensure baby is kept warm, feedings continued

C. CONDITION AT THE TIME OF REFERRAL

Consciousness: Conscious ☐ Semiconscious ☐ Unconscious ☐ Pulse: ________/min BP: _____________mmHg
Temperature: _____ ^{0}C Respiration: _______/min Urine output: ________cc/hr Other specify______________________

D.REFERRAL DETAILS

Date diagnosis made ☐☐☐☐☐☐ Time diagnosis made ☐☐ hrs ☐☐ mins

Diagnosed by SN ☐ MO ☐ Other ☐ Staff name ___________________________ Signature ________________________

Details of facility to which the woman is referred

Name _________________Location _____________________District ___________________________Taluka ____________________

Type CHC ☐ TH ☐ DH ☐ Private ☐ Other ☐ (specify)______________________________

Person to contact ____________________________Telephone # ____

Date left PHC ☐☐☐☐☐☐ Time left PHC ☐☐ hrs ☐☐ mins

Type of vehicle used for transportation 108 ☐ Ambulance Govt ☐ Ambulance Pvt ☐ Other specify ☐ _______________________

Outcome of referral Discharged healthy ☐ Died ☐ Date ☐☐☐☐☐☐ Time ☐☐ hrs ☐☐ mins

ಸುಕ್ಷೇಮ sukshema Improved Maternal, Newborn & Child Health

COMPLICATION CASE SHEET G

For initial management and referral of newborns with complications

Send a copy of the case sheet along with this sheet to the referral facility

A.BACKGROUND INFORMATION

District ______________________ Taluka ____________________ PHC Location ______________

Name______________________________ Thayi card number ☐☐☐☐☐☐☐☐

Address ____

B.SPECIFIC DIAGNOSIS AND INITIAL MANAGEMENT

Diagnosis (Tick as applicable a-f)

a. ☐ Preterm birth < 37 weeks gestation

b. ☐ Low birth weight requiring referral (less than 1800 gms)

c. ☐ Asphyxia (Tick as appropriate)
- ☐ Not breathing after 30 secs bag and mask
- ☐ APGAR < 7 at 5 mins

d. ☐ Infection / Sepsis (Tick as appropriate)

☐ Respiratory distress
- ☐ Grunting ☐ RR >60 breaths/min
- ☐ Severe chest indrawing ☐ Nasal flaring

☐ Temperature instability
- ☐ Temp $\geq$ 37.5 ^{0}C or $\leq$ 35.5 ^{0}C

☐ Skin problems - 10 or more skin pustules or big boil

☐ Abnormal activity
- ☐ Convulsions ☐ Lethargic or unconscious
- ☐ Little movement ☐ Floppy or stiff baby

☐ Feeding problems – unable to feed

☐ Blue or pale baby

e. ☐ Birth anomalies (specify) ________________

f. Others (Tick as appropriate)
- ☐ Jaundice ☐ Bleeding from any site
- ☐ No urine after 48 hrs ☐ Abdominal distension
- ☐ Persitent vomiting or diarrhea
- ☐ Other (specify)…………………………………

THESE ARE ALL NEWBORN EMERGENCIES

Initial management at the PHC (Tick when management done)

- ☐ Call and determine the nearest facility for special newborn care
- ☐ Arrange transport
- ☐ Keep the baby warm, preferably using KMC

Give fluids, either
- ☐ Breast milk/breastfeeding or
- ☐ 5ml/kg expressed breast milk by palladai or
- ☐ 5ml/kg expressed breast milk by OG tube or
- ☐ 5ml/kg of 10% Dextrose by palladai or
- ☐ 5ml/kg of 10% Dextrose by OG tube

☐ Give ventilation with bag and mask, and oxygen if necessary

☐ Chest compressions given (if trained)

☐ During transport, continue feeding and bag and mask, if necessary

If you suspect infection give following antibiotics
- ☐ Ampicillin 50 mg/kg IM and
- ☐ Gentamicin 5 mg/kg IM

Time antibiotics given ☐☐ hrs ☐☐ mins

Reason if not given ______________________________

☐ Check baby received vitamin K and if not, then give as follows
- ☐ 1 mg if weight > 1500 gms
- ☐ 0.5 mg if weight ≤ 1500 gms
- ☐ Given before

C. CONDITION AT THE TIME OF REFERRAL

Activity Active ☐ Lethargic ☐ Irritable ☐ Heart rate ______/min Respiratory rate ______/min

Temperature: _____ ^{0}C Other specify ________________________________

D. REFERRAL DETAILS

Date diagnosis made ☐☐☐☐☐☐ Time diagnosis made ☐☐ hrs ☐☐ mins

Diagnosed by SN ☐ MO ☐ Other ☐ Staff name ______________________ Signature ____________________

Details of facility to which the woman is referred

Name _______________Location ____________________District _________________________Taluka _________________

Type CHC ☐ TH ☐ DH ☐ Private ☐ Other ☐ (specify)___________________________

Person to contact ___________________________Telephone # ____

Date left PHC ☐☐☐☐☐☐ Time left PHC ☐☐ hrs ☐☐ mins

Type of vehicle used for transportation 108 ☐ Ambulance Govt ☐ Ambulance Pvt ☐ Other specify ☐ ______________________

Outcome of referral Discharged healthy ☐ Died ☐ Date ☐☐☐☐☐☐ Time ☐☐ hrs ☐☐ mins

Project Sukshema Department of Health, Government of Karnataka National Rural Health Mission

COMPLICATION SHEET H

For initial management and referral of other maternal complication

Send a copy of the case sheet along with this sheet to the referral facility

A.BACKGROUND INFORMATION

District ______________________ Taluka __________________ PHC Location _________________

Name ________________________ Thayi card number ☐☐☐☐☐☐☐☐

Address ____

Contact number ☐☐☐☐☐☐☐☐☐☐

Date of admission ☐☐☐☐☐☐☐☐ Time of admission ☐☐ hrs ☐☐ mins

Date of delivery ☐☐☐☐☐☐☐☐ Time of delivery ☐☐ hrs ☐☐ mins

Date of referral ☐☐☐☐☐☐☐☐ Time of referral ☐☐ hrs ☐☐ mins

B. PARTICULARS OF REFERRAL

Provisional diagnosis: ____

Chief complaints: ____

____

Summary of management (Pre-referral treatment/ procedures):

____

____

____

Investigations: ____

____

C. CONDITION AT THE TIME OF REFERRAL

Consciousness: Conscious ☐ Semiconscious ☐ Unconscious ☐ Pulse: _______/min BP: ___________mmHg

Temperature: _____⁰ C Respiration: ______/min Others specify ___________________________

D. REFERRAL COMMUNICATION AND TRANSPORT

Date diagnosis made ☐☐☐☐☐☐ Time diagnosis made ☐☐ hrs ☐☐ mins

Diagnosed by SN ☐ MO ☐ Other ☐ Staff name ______________________ Signature ____________________

Details of facility to which the woman is referred

Name ______________Location ____________________District _________________________Taluka __________________

Type CHC ☐ TH ☐ DH ☐ Private ☐ Other ☐ (specify)_____________________________

Person to contact ___________________________Telephone # ____

Date left PHC ☐☐☐☐☐☐ Time left PHC ☐☐ hrs ☐☐ mins

Type of vehicle used for transportation 108 ☐ Ambulance Govt ☐ Ambulance Pvt ☐ Other specify ☐ ______________________

Outcome of referral Discharged healthy ☐ Died ☐ Date ☐☐☐☐☐☐ Time ☐☐ hrs ☐☐ mins
