# Supplementary material for: Nurse Mentors to Advance Quality Improvement in Primary Health Centers: Lessons From a Pilot Program in Northern Karnataka, India
Source: Glob Health Sci Pract. 2015 Dec 15;3(4):660–75. doi: 10.9745/GHSP-D-15-00142 (PMC4682589; doi:10.9745/GHSP-D-15-00142)
Supplement: Supplementary Material [file GHSP-D-15-00142_15-00142-Fischer-Supplementary-Material-Self-Assessment-Checklists.pdf]

Self-assessment guides

Project Sukshema – 24/7 PHC - Self Assessment guide		
	**A.Clients' Rights to Safe and Competent Care**	Tick (✓) if Yes or fill 'X'
1.	Do PHC clinical providers fully and accurately document the woman's history and details of lab tests, vaginal and pelvic exams on client case sheets?	
2.	Do PHC clinical providers fully and accurately document all complications (diagnosis and management) on the complications sheets?	
3.	Do staff monitor and document the diagnosis of active labour and the process of labour fully and accurately on partographs?	
4.	Do staff monitor all women and newborns post delivery and document on the case sheets?	
5.	Do staff ensure that women in 2nd stage of labour are never left alone?	
6.	Do all staff know how to assess, diagnose and manage women in shock?	
7.	Do all staff know how to, and practice Active Management of third stage of labour (AMTSL) including appropriate use of oxytocin for delivery of the placenta (but NEVER for labour augmentation)?	
8.	Are all staff able to recognize severe anaemia and refer when necessary?	
9.	Are all staff able to assess, diagnose and manage:<br>Prolonged rupture of membranes (PROM), obstructed and/or prolonged labour?	
10.	Are all staff able to assess, diagnose and manage antepartum and postpartum haemorrhage?	
11.	Are all staff able to take BP and assess, diagnose and manage pregnancy induced hypertension, pre-eclampsia and eclampsia?	
12.	Do all staff know to, and practice administration of magnesium sulphate for convulsions?	
13.	Are all staff able to assess, diagnose and manage pre-term labour or preterm prelabour rupture of membranes?	
14.	Do all staff know and practice administration of corticosteroids according to guidelines (how much, when and where) in pre-term labour for the prevention of neonatal respiratory distress?	
15.	Are all staff able to assess, diagnose and manage the different causes of maternal sepsis?	
16.	Do all staff know not to do episiotomies unless absolutely necessary and not to augment labour at a 24/7 PHC?	
17.	Do all staff know how to assess, diagnose and manage foetal distress?	
18.	Do all staff know how to, and practice?<br>Immediate neonatal care, including bag/mask ventilation if resuscitation required; cord care; eye care;thermal protection and kangaroo mother care; immediate breastfeeding support; appropriate vaccinations and vitamin K for newborns before discharge	
19.	Are all staff able to assess, diagnose and manage prematurity and low birth weight?	
20.	Are all staff able to assess, diagnose and manage neonatal asphyxia?	
21.	Are all staff able to assess, diagnose and manage newborn sepsis?	
22.	Do all staff know how to help a woman to breastfeed and how to manage problems?	
23.	Do all staff know how, and practice all the steps, and timings of postpartum and what danger signs to look for? Newborn monitoring and what danger signs to look for?	
24.	Do staff, before discharging postpartum clients, know how what advice to give new mothers about themselves and the newborn?	
25.	Do staff know how to initiate a referral and communicate with the closest higher level for emergency obstetric or newborn services?	

Comments

____

____

Project Sukshema – 24/7 PHC - Self Assessment guide

	**B.Staff's Right to Supplies, Equipment and Infrastructure**	Tick (✓) if Yes or fill 'X'
1.	Is there a good system for repair/maintenance of equipment, so services are not interrupted?	
2.	Is there a functional system for monitoring and ordering of drugs and supplies so services are not interrupted?	
3.	Are the following available (in working order) in the exam room and labour room?<br>Exam table<br>Adjustable light<br>BP apparatus<br>Foetoscope<br>Speculum; Forceps (Cheatle and Sponge)<br>Case sheets<br>Registers<br>Partographs	
4.	In the labour room, is there a newborn corner with:<br>Table or flat surface, Blanket or towel for warmth, Thermometer<br>Baby weigh scale<br>Radiant warmer<br>Neonatal ambu bag and mask (with different sized masks)<br>Oxygen, Nasal catheter, Mucus extractor, Cord ties/clamps<br>Clean dry blankets<br>Infant Stethoscope	
5.	Are all the following drugs available or readily accessible 24/7?<br>1. Anti-convulsants (magnesium sulphate) and calcium gluconate for drug reaction or diazepam (can be used for severe pre-eclampsia and eclampsia)<br>2. Uterotonics (oxytocin or misoprostol) for AMTSL (NOT for augmentation)<br>3. Anti PPH uterotonic (oxytocin)<br>4. Antibiotics (gentamycin, ampicillin, metronidazole) for sepsis/ sepsis prophylaxis<br>5. Cloxacillin, Erythromycin for breast abscess<br>6. Diazepam (for removal of retained placenta)<br>7. Methergin, carboprost or misoprostol for atonic uterus and delayed PPH<br>8. IV fluids/Ringer lactate<br>9. Anti-hypertensives (nifedipine or hydralazine)<br>10. Corticosteroids (betamethasone or dexamethasone)<br>11. Chlorhexidine<br>12. Vitamin K<br>13. All needed vaccines	
6.	Are all infection prevention materials available?<br>Soap and running water<br>Surgical gloves<br>Utility gloves for cleaning instruments<br>Buckets with chlorine (0.5%, WITH LIDS), renewed every day<br>Sharps disposal containers<br>Waste buckets for medical and other waste	
7.	Is the pharmacy always ready, stocked,<br>open for clients with complications 24/7?<br>Is there always an emergency tray with all needed drugs in the labour room?	

8.	Is the lab always ready, with staff and all reagents and equipment needed to perform basic tests 24/7?<br>Haemoglobin<br>Urinalysis for sugar<br>Urinalysis for proteins<br>Urinalysis for bacterial infections<br>HIV tests<br>RPR/VDRL<br>HBsAg<br>If the lab is closed, are staff able to access HIV rapid tests and urine dipsticks for protein testing?	
9.	Is the instrument processing area fully equipped at all times with? Working autoclave or boiler,Deep sink and running water, Brushes, Detergent, Chlorine, Basins, Utility gloves	
10.	Does the PHC have a deep pit for burying waste?	

Comments

____

____

____

Project Sukshema – 24/7 PHC - Self Assessment guide<br>ಸುಕ್ಷೇಮ<br>sukshema<br>Improved Maternal, Newborn & Child Health		
	**C. Clients' Rights to Access to Services and Continuity of Care**	Tick (✓) if Yes or fill 'X'
1.	Do clients have access to labour and delivery services 24 hours a day, 7 days a week available?<br>• Qualified staff available to offer labour and delivery services (doctors, nurses, labs staff, pharmacist)?<br>• Are there always supplies available to provide labour and delivery services?<br>• Is there always emergency transportation service available for referrals?	
2.	Do staff minimize procedural and financial barriers to care for clients (e.g. requirement that clients obtain a male family member's permission to receive services, have easy access to services without having to pay or purchase supplies or drugs)?	
3.	Are all obstetric clients in labour evaluated within 15 minutes of arrival at your facility?	
4.	Do ANMs and ASHAs appropriately refer women with or without complications to the PHC?	
5.	Do staff encourage all women to stay in the PHC for 48 hours post delivery?	
6.	Are all nursing staff able to do the following always?<br>• Set up an IV drip<br>• IM Injection of oxytocin for AMTSL and PPH<br>• Intravenous antibiotic administration<br>• Inject IM magnesium sulphate<br>• Repair a simple tear	
7.	Are all necessary following things available?<br>• Supplies (e.g. gloves, IV fluids, oxygen)<br>• Drugs (e.g. antibiotics, anti-hypertensives, anti-convulsants, uterotonics)<br>• Equipment available 24/7 without barriers (e.g. locked doors or cabinets or unavailable keys)	
8.	Does the facility have all needed recording sheets (case sheets and registers) and do all staff know what is expected of them in terms of recording?	
10.	Do staff do the following for women or babies who need referral??<br>1. Provide or arrange transport within one hour of identification of need to refer?<br>2. Communicate with the referral facility to inform them about the referral?<br>3. Complete all paperwork about the person being referred including history, details of tests performed, drugs given, diagnosis?<br>4. Know about and record the outcome of the person referred?<br>5. Ensure the person referred is also then referred back to the ANM and ASHA in her community?	

Comments:

____

____

Project Sukshema – 24/7 PHC - Self Assessment guide		
	**D.Clients' Rights to Infection-free Services**	Tick (✓) if Yes or fill 'X'
1.	Do staff ensure they do not do unnecessary procedures that can increase the risk of infection (e.g. too frequent vaginal exams, routine IV, routine episiotomy)	
2.	Do staff initiate treatment of all suspected or confirmed sepsis with ampicillin, gentamycin and metronidazole?	
3.	Do staff wash their hands with soap and running water?<br>• Before each clinical procedure<br>• After each clinical procedure<br>• Before and after each client contact	
4.	Does your facility have the following protective wear for staff? Aprons, Caps, Eyewear, Face masks, Shoe covers/boots, Sterile gloves	
5.	Do staff change gloves if they become contaminated (i.e. between clients and with the same client if glove gets contaminated)?	
6.	Between deliveries, do staff wipe the tables and contaminated surfaces in every room with 0.5% chlorine solution?	
7.	Do staff know how to, and follow all correct steps of instrument processing? Instruments decontaminated immediately after use in 0.5% chlorine solution, Washed in running water, Sterilization or HLD; Drying, Storage	
8.	Do staff always ensure proper disposal of sharps in a puncture proof container?	
9.	Do staff segregate and dispose of all medical waste (including bodily waste, swabs and sharps containers) according to guidelines?	
10.	Are the labour room, ward and toilets as clean as possible?	

Comments

____

____

Project Sukshema – 24/7 PHC - Self Assessment guide

	E.**Staff's Right to Information, Training and Development**	Tick (✓) if Yes or fill 'X'
1.	Are current NRHM and SBA / NSSK guidelines available and accessible to all staff	
2.	Are all staff trained to manage labour and deliveries as per SBA standards?	
3.	Are all staff trained to deal with pre-referral management of complications of labour and newborns?	
4.	Is a sufficient number of nursing staff trained to take<br>• BP<br>• insert a Foley's catheter<br>• administer IV fluids<br>• administer antibiotics<br>• administer oxytocin<br>• administer magnesium sulphate<br>• administer corticosteroids	
5.	Is a sufficient number of staff trained, and ALWAYS available, to perform neonatal resuscitation?	
6.	Are all staff trained in infection prevention?	
7.	Are all nursing staff trained and able to do:<br>1. urine dipstick tests for protein;<br>2. HIV rapid tests	
8.	Does the pharmacist:<br>A. Know what drugs are essential for labour<br>B. Know what drugs are essential for delivery<br>C. Ensure all emergency drugs are in the labour room during labour?<br>D. Does s/he (and nurses) know what drugs (e.g. oxytocin) require cold chain?	
9.	Do all staff know the list of possible hospital options for referral of women who have complications that cannot be managed at the PHC and know how to refer?	

Comments

____

____

____

Project Sukshema – 24/7 PHC - Self Assessment guide

	**F. Clients' Rights to Privacy, Confidentiality, Dignity, Comfort and Expression of Opinion**	Tick (✓) if Yes or fill 'X'
1.	Does each of the following areas offer visual and auditory privacy from other clients and staff?<br>1. Examination rooms<br>2. Labour and delivery rooms<br>3. Counselling areas	
2.	Do staff keep body exposure to a minimum duration and extent during vaginal exams and delivery?	
3.	Do staff refrain from talking about clients with people who are not directly involved in their care?	
4.	When client records are not in use, do staff store them in a secure place (e.g. with access strictly limited to authorized staff)?	
5.	Is each of the following areas always clean and comfortable?<br>1. Examination rooms<br>2. Maternity wards<br>3. Labour and delivery rooms<br>4. Toilets<br>5. Waiting areas	
6.	Does your PHC provide the following for clients and those who come with them?<br>1. Clean drinking water<br>2. Clean toilets<br>3. Nourishing food	
7.	Do staff always provide pain medication for those that need it, and emotional support during labour and in the postpartum period?	
8.	Do staff provide counselling and emotional support to clients and their families in the case of complications such as?<br>A. maternal or foetal death<br>B. severe morbidity<br>C. foetal abnormalities	

Comments

____

____

Project Sukshema – 24/7 PHC - Self Assessment guide		
	**G.Clients' Rights to Information and Informed Choice**	Tick (✓) if Yes or fill 'X'
1.	When clients with complications have been stabilized, do staff inform all clients and their families about the diagnosis, need for treatment or procedures and possible outcomes?	
2.	Before discharging postpartum women and babies, do staff know about, and provide information on family planning?	
3.	Before discharging postpartum women and babies, do staff know about, and provide information on infant feeding and the importance of exclusive breastfeeding for 6 months?	
4.	Before discharging postpartum women and babies, do staff know about, and provide information on danger signs in the mother?	
5.	Before discharging postpartum women and babies, do staff know about, and provide information on danger signs in the newborn?	
6.	Before discharging postpartum women and babies, do staff provide information on how to contact the ANM and ASHA in her area?	
7.	When a client is referred, do staff explain why it is important to go to a facility that can manage the complication?	

Comments

____

____

____

____

Project Sukshema – 24/7 PHC - Self Assessment guide

	**H.Staff's Right to Facilitative Supervision and Management and safe and secure work environment**	Tick (✓) if Yes or fill 'X'
1.	Do off-site supervisors or technical experts visit the facility regularly and do the following?:<br>1. Assess services<br>2. Recognize success<br>3. Work with staff to solve problems<br>4. Provide constructive feedback on clinical skills<br>5. Provide constructive feedback on general management of services<br>6. Provide information on government MNCH and SBA guidelines<br>7. Provide new knowledge through mini-trainings<br>8. Review management of complications of labour with you	
2.	Do you together, as staff, regularly assess the services you are providing (such as observing your infection prevention practices)?	
3.	Do you together, as staff, review emergency protocols?	
4.	Do you together, as staff, review the following to assess your progress?<br>• case sheets<br>• registers and records	
5.	Does PHC management meet with staff regularly to discuss problems?	
6.	Does PHC management encourage staff to work together as a team, respecting all staff and their roles (including ancillary staff, nurses, pharmacists, lab people, doctors, record keepers)?	
7.	Does everyone have clear job descriptions and know their responsibilities?	
8.	Are referral protocols and referral facility contact information readily available and protocols understood by all staff?	
9.	Is there a system for ensuring transport to a referral facility for clients 24 hours a day, 7 days a week that ensures transport within one hour?	
10.	Does the PHC keep and post on the wall a statistics chart with monthly information on?<br>• Number of deliveries<br>• Number of referrals	
11.	Are all complicated cases and cases with poor outcomes (i.e. maternal or neonatal morbidity or mortality) regularly reviewed at staff meetings?	
12.	Does PHC management share information about the performance of the PHC (e.g. service statistics, findings from district quality team visits, etc.)?	
13.	Are there well-functioning processes for determining staff schedules, managing coverage during leaves and absences, filling vacant positions?	
14.	Are there well-functioning processes for determining staff schedules, managing coverage during leaves and absences, filling vacant positions?	
15.	Are there adequate levels of security at the facility so that staff and patients feel safe at all hours?	
16.	Are there supportive systems and means of redressal in place if staff experience gender based violence or harassment at work?	

Comments

____

____
